# A geological carbon cycle sink hosted by ocean crust talus breccias

**DOI:** 10.1038/s41561-025-01839-5

**Published:** 2025-11-24

**Authors:** Rosalind M. Coggon, Elliot J. Carter, Lewis J. C. Grant, Aled D. Evans, Christopher M. Lowery, Damon A. H. Teagle, Pamela D. Kempton, Matthew J. Cooper, Claire M. Routledge, Elmar Albers, Justin Estep, Gail L. Christeson, Michelle Harris, Thomas M. Belgrano, Jason B. Sylvan, Julia S. Reece, Emily R. Estes, Trevor Williams

**Affiliations:** 1 School of Ocean and Earth Science, National Oceanography Centre Southampton, University of Southampton, Southampton, UK; 2 School of Life Sciences, Keele University, Newcastle-under-Lyme, UK; 3 University of Texas Institute for Geophysics, University of Texas at Austin, Austin, TX USA; 4 Department of Geology, Kansas State University, Manhattan, KS USA; 5 Institute of Geosciences, Christian-Albrechts-University of Kiel, Kiel, Germany; 6 Section of Geophysics, Alfred Wegener Institute, Helmholtz Centre for Polar and Marine Research, Bremerhaven, Germany; 7 School of Earth and Sustainability, Northern Arizona University, Flagstaff, AZ USA; 8 Marine Geology and Geophysics, National Science Foundation, Alexandria, VA USA; 9 School of Geography, Earth and Environmental Sciences, University of Plymouth, Plymouth, UK; 10 UCD School of Earth Sciences, University College Dublin, Dublin, Ireland; 11 Department of Oceanography, Texas A&M University, College Station, TX USA; 12 Department of Geology and Geophysics, Texas A&M University, College Station, TX USA; 13 International Ocean Discovery Program, Texas A&M University, College Station, TX USA; 14 Present Address: Marine Geology and Geophysics, National Science Foundation, Alexandria, VA USA

**Keywords:** Geochemistry, Tectonics, Volcanology, Carbon cycle, Petrology

## Abstract

Calcium carbonate precipitation in ageing ocean crust sequesters carbon dioxide dissolved in seawater through seafloor weathering reactions, influencing atmospheric CO_2_ concentrations on million-year timescales. However, this crustal carbon sink, and the extent it balances CO_2_ degassing during crustal formation at mid-ocean ridges, remain poorly quantified due to limited sampling of the vast ridge flanks where CO_2_ uptake continues for millions of years. Here we quantify the carbon sink hosted within talus breccias that accumulated through mass wasting 61 million years ago during rift faulting at the slow spreading Mid-Atlantic Ridge, cored during International Ocean Discovery Program South Atlantic Transect Expedition 390. After 40 million years of carbonate cementation, these breccias contain ~7.5 wt% seawater-derived CO_2_, 2 to 40 times more than previously cored upper crust. Our estimates of talus breccia abundance based on fault geometries indicate that talus formed at slow-spreading ridges can accommodate a CO_2_ sink equivalent to a large proportion of the CO_2_ released during accretion of the underlying crust. The proportion of plate divergence accommodated by faulting, and hence talus abundance, increases nonlinearly with decreasing spreading rate. Consequently, past variations in spreading rate may have impacted the balance between ocean crust CO_2_ release and uptake in Earth’s carbon cycle.

## Abstract (web summary)

Mass-wasting deposits that accumulated against mid-ocean ridge faults have high porosity in which calcium carbonate precipitated, storing seawater carbon dioxide, as revealed by cores of a 61-million-year-old seafloor talus deposit.

## Main

The formation of new ocean crust at mid-ocean ridges (MORs), its subsequent ageing over millions of years as it traverses the ocean basins and eventual subduction into the mantle is an integral component of the geological carbon cycle. Basaltic magma degassing during MOR volcanism releases large volumes of CO_2_ to the oceans and atmosphere, with the modern global flux estimated to be ${1.32}_{-0.85}^{+0.77}$ × 10^12^ mol yr^−1^ (ref. ^1^). Subsequent precipitation of calcium carbonate (CaCO_3_) minerals during circulation of seawater-derived hydrothermal fluids through the ocean crust sequesters CO_2_, which is transported towards subduction zones as the crust ages^2–4^. This carbon sink can be quantified from sections of hydrothermally altered ocean crust that provide time-integrated records of their geochemical exchanges with hydrothermal fluids. The fluxes of CO_2_ from and to the ocean crust remain poorly quantified but are often assumed to be in balance^5^, although evidence suggests they are not^2,6–8^. Whether the formation and evolution of the ocean crust influenced past atmospheric CO_2_ concentrations, and hence climate, remains debated^2–5,8,9^, principally due to imprecise knowledge of the conditions, extent, depth and duration of hydrothermal CaCO_3_ precipitation within the ageing ridge flanks.

Given their vast extent globally, the hydrothermal fluid flux through ridge flanks is many orders of magnitude greater than that through high-temperature (≤400 °C) axial systems^10^. Conductive heat-flow studies indicate advection of heat by low-temperature (<100 °C) hydrothermal fluids persists across ridge flanks for 65 Myr on average (for example, refs. ^11–13^). However, fluid flow can occur at any crustal age given sufficient hydrologic driving forces. The extent and duration of ridge flank fluid flow are therefore affected by various parameters including basement topography, volcanic stratigraphy and lava morphology, the extent to which hydrothermally sealed fractures (veins) are re-cracked and the composition, thickness and completeness of sediment cover (for example, refs. ^14–16^), many of which are strongly influenced by spreading rate. Scientific ocean drilling provides the only means to sample ocean crust across the sedimented ridge flanks. However, due to unrepresentative sampling of upper ocean crust with respect to basement age, spreading rate and sediment thickness^17,18^, there was a dearth of drill core from middle-aged (20–100 Ma) and slow-spread crust through which heat-flow studies and crustal architecture suggest prolonged circulation of seawater should occur on ridge flanks.

Newly acquired cores of 7 to 61 Ma slow-spread crust drilled in the western South Atlantic along the South Atlantic Transect (SAT) during International Ocean Discovery Program (IODP) Expeditions 390 C, 395E, 390 and 393 provide a unique opportunity to understand the role of ageing ocean crust in long-term biogeochemical cycles and the influence of spreading rate on hydrothermal alteration through comparison with other well-characterized sites^17^. Carbonate-cemented talus breccia cores from IODP Site U1557, located on 60.7-Ma crust on the western flank of the southern Mid-Atlantic Ridge, represent a previously underappreciated sink in the global carbon cycle hosted by slow-spread crust. This sink occurs as a direct consequence of the impact of slow-spreading rates on crustal architecture.

At slow-spreading ridges >10% of the plate divergence is accommodated by tectonic strain through normal faulting, resulting in a well-defined axial valley 0.8–1.6-km deep and 5–25-km wide (for example, refs. ^19–22^). The relief, slopes and seismicity of the normal fault scarps promote erosion and mass wasting, resulting in accumulation of porous basaltic talus breccias against the axial valley walls^23,24^. These faults remain active as the crust spreads away from the axis resulting in rough horst and graben basement topography on the ridge flanks^25^. Coring at Site U1557 recovered the first talus breccias from a middle-aged slow-spreading ridge flank, revealing that through carbonate precipitation and cementation over tens of millions of years, these clastic deposits sequester large quantities of dissolved seawater carbon. Here we describe the hydrothermal cementation of Site U1557 talus breccias, quantify their CO_2_ uptake over at least 40 million years and extrapolate these observations to estimate the carbon sink that talus breccias on slow-spread crust may constitute.

## SAT crustal architecture

**Fig. 1:**
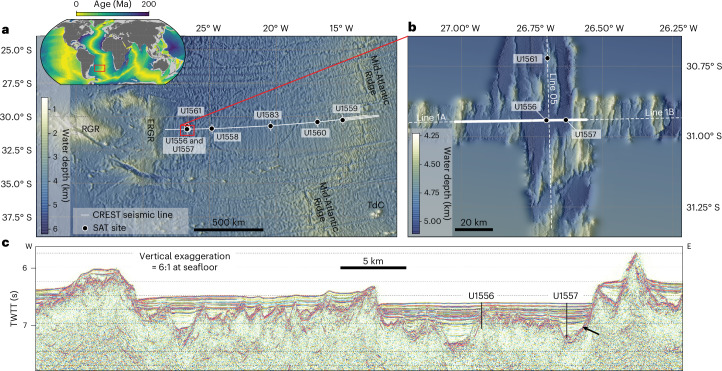
Location and tectonic setting of the SAT drill sites. **a**, Bathymetry^47^ of SAT area in the South Atlantic Ocean (adapted from ref. ^17^), showing the locations of the SAT drill sites (black circles); inset shows the location of the SAT area on a map of seafloor age^48^. **b**, Bathymetry^47^ of the ~61-Ma SAT study area, where sites U1557 and U1556 are located in the same sediment-filled basin^17^. White lines, CREST seismic reflection profiles^49^; RGR, Rio Grande Rise; ERGR, eastern Rio Grande Rise; TdC, Tristan de Cunha. **c**, CREST seismic reflection profile^49^, parallel to the SAT, showing the locations of SAT Site U1557 adjacent to a Mid-Atlantic Ridge-parallel fault and Site U1556 on the adjacent faulted basement high; portion of the CREST line shown indicated by the solid white line in the study area map **b**. Black arrow indicates the sediment–basement interface. TWTT, two-way travel time. The sediment-free, or thinly sedimented, exposed basement ridges ~15 km west and ~ 5 km east of Site U1557 allow fluids in and out of the basement, with lateral channelling through the basement between these outcrops likely, as observed through upper crustal aquifers elsewhere^50,51^.

The SAT drilling recovered complete sedimentary sections and up to 340 m of the underlying oceanic crust along a crustal flowline across the western flank of the slow- to intermediate-spreading (13–31 mm yr^−1^ half rate^26^) Mid-Atlantic Ridge at ~31° S (ref. ^17^) (Fig. 1). Before drilling, the Crustal Reflectivity Experiment Southern Transect (CREST) conducted a detailed geophysical survey of crust formed 0–70 million years ago (Ma) along the ~1,400 km SAT crustal flowline^26–28^. Seismic imaging revealed a well-developed ridge–basin seafloor texture outside the normal fault-bounded axial valley, comprising sediment-filled fault-bounded basins (up to ~650-m deep and 2–4 km across) sub-parallel to the ridge axis and common small seamounts^27^. Sediment-free or thinly sedimented exposed basement outcrops that may allow seawater and ridge flank hydrothermal fluids to flow in and out of the crust are abundant along the CREST survey line (for example, Fig. 1c). As the crust is never fully sealed by sediment^28^, there may be long-lived (>65 Myr) and ongoing connection between the ocean and uppermost crust. This rough topography contrasts with reference sections of faster-spread ocean crust that have been the focus of investigation in recent decades, where high sedimentation rates (for example, refs. ^29,30^) promoted sealing of the crust from the ocean leading to anomalously high basement temperatures and earlier cessation of hydrothermal circulation^31^.

## Site U1557 carbonate-cemented talus breccias

**Fig. 2:**
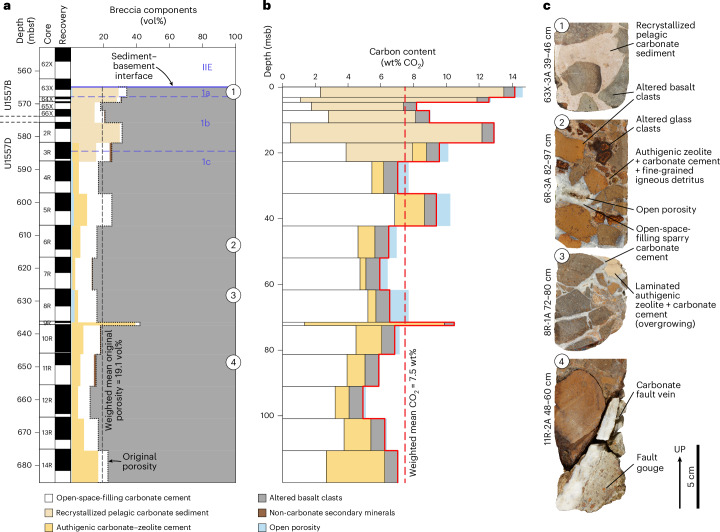
Site U1557 talus breccia stratigraphy and carbon content. **a**, Composite U1557 stratigraphy, comprising Holes U1557B and U1557D, showing the variations in abundance (vol%) of breccia components downhole. Depth is given in both metres below seafloor (mbsf) and metres sub-basement (msb). Black bars indicate degree of recovery in each core interval. The solid blue line indicates the sediment–basement interface, and dashed blue lines and numbers indicate lithological subunits. The original porosity is outlined with black dots and the mean total original porosity of the cored deposit, weighted by core interval lengths to normalize to core recovery (Supplementary Table 1), is indicated by the dashed black line. Numbers in circles indicate the depths of core photographs in **c**. **b**, Total carbon content (wt% CO_2_) of each core (solid red line), calculated from the CO_2_ concentrations, volume proportions and densities of the carbonate-bearing components, following equation (2), with the contribution of each breccia component indicated by the coloured bars. The mean CO_2_ content of the breccia, weighted by core interval lengths to normalize to core recovery (Supplementary Table 1), is indicated by the red dashed line. Blue bars indicate the additional CO_2_ capacity of the breccia, if all open porosity was filled with CaCO_3_ cement. **c**, Photographs of selected core pieces^17^ illustrating key features: (1) altered basalt clasts in a recrystallized pelagic carbonate sediment matrix; (2) typical carbonate-cemented breccia; (3) breccia with authigenic zeolite + carbonate cement overgrowing sparry carbonate cement; (4) carbonate fault vein cutting through breccia at high angle and underlain by fine-grained greenish fault gouge.

Given considerable variations in sediment thickness and continuity along the SAT^26,27^, Expedition 390 occupied two sites on ~61-Ma crust to investigate the variability in duration and extent of hydrothermal alteration due to rugged basement topography at a given crustal age. The 61.2-Ma crust at Site U1556 is overlain by ~278 m of sediment, whereas Site U1557, ~6.5 km to the east, is located on 60.7-Ma crust in a more thickly sedimented (~564 m) portion of the same local sediment basin^17^ (Fig. 1). The upper basement cored at U1556 comprises a ~340-m volcanic sequence of pillow lavas, hyaloclastite breccias and massive flows typical of slow-spread crust. In contrast, at Site U1557 the upper basement comprises a basaltic talus breccia deposit, with Hole U1557B capturing the sediment–basement interface and Hole U1557D penetrating 120 m into the deposit, without reaching intact lavas (Fig. 2). The talus is interpreted to have formed against the adjacent uplifted basement fault block^17^. Middle Palaeocene planktic foraminifera and calcareous nannoplankton in the sediments directly overlying the breccia are indicative of Zones P4a (60.54–60.76 Ma) and CNP7 (60.76–61.17 Ma), respectively^17,32^, indicating the breccia accumulated soon after accretion of the underlying lavas, probably during uplift on axial valley wall bounding faults.

The U1557 breccia is largely clast supported, comprising centimetre-scale fragments of pillow basalt displaying a range of pre- and post-brecciation alteration styles and millimetre- to centimetre-scale fragments of altered volcanic glass. Between these clasts was considerable primary porosity equivalent to ~12–42 vol% of the recovered cores, averaging >19 vol% (Fig. 2 and Supplementary Table 1). Consequently, the breccia deposit had high interconnected porosity with a large surface area for fluid–rock reaction and substantial accommodation space for hydrothermal precipitates. This macropore space is now variably filled by sparry carbonate cement, fine-grained clastic volcanic material, microcrystalline authigenic zeolite + carbonate cement and/or pelagic sediment, with millimetre- to centimetre-scale open porosity still common throughout much of the core (<2.4 vol%; Fig. 2).

Despite the rubbly nature of the original talus pile, there was good core recovery (averaging 63%; Fig. 2 and Supplementary Table 1). Individual core pieces <122 cm long and a maximum core recovery of 97%, despite comprising centimetre-scale basaltic clasts, reflect the robustness of hydrothermal cementation. Sparry calcite cement is ubiquitous, typically growing into open space, with a dog-toothed habit. Sparry calcite growths covering the ends of many core pieces indicate there are voids wider than the 6-cm diameter core, and consequently, the in situ formation-scale porosity is probably underestimated based on description of recovered material alone. In the lower part of the cored hole (>75 metres sub-basement; msb), calcite cements have a sugary texture, and the remnant porosity is lower^17^. Altogether, these open-space-filling carbonate cements make up 1.1 to 14.6 vol% of the recovered cores with a mean abundance of 8.7 ± 3.2 vol% (±1*σ*), weighted by core interval lengths to normalize for variable core recovery (Fig. 2 and Supplementary Table 1).

Low-temperature hydrothermal alteration of the basaltic breccia clasts is also ubiquitous, manifest in the formation of secondary minerals, which include calcite in addition to clays, iron oxyhydroxides and zeolite. These carbonate-bearing assemblages replace olivine and plagioclase phenocrysts and groundmass, and fill vesicles and fractures to form veins^17^.

In the uppermost breccia cores, from immediately below the sediment–basement interface, buff to pale peach recrystallized microfossil-bearing pelagic sediments form the breccia matrix and crystalline cement is rare. Pelagic sediment is absent deeper than ~20 m into the breccia, but a microcrystalline cement similar in appearance to the sediment matrix is ubiquitous below this depth. This cement comprises an authigenic mixture of phillipsite zeolite and carbonate, providing an additional hydrothermal carbon sink component within the breccia.

**Fig. 3:**
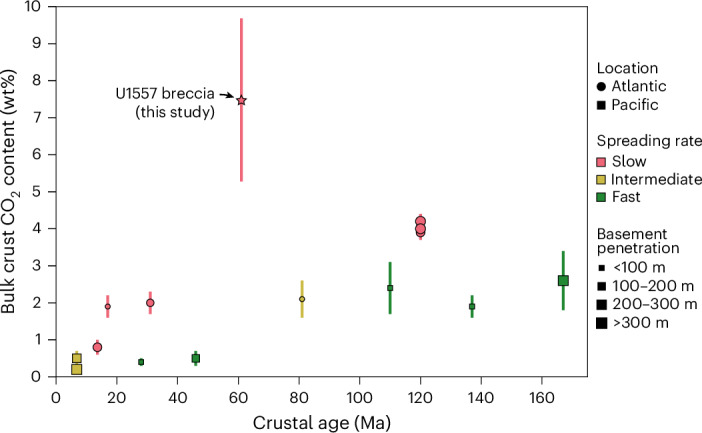
Bulk carbon dioxide contents of upper ocean crustal sections versus crustal age. The mean CO_2_ content of the U1557 breccia cores (star), weighted by core interval lengths to normalize to core recovery, is compared to bulk CO_2_ contents of previously drilled sections of intact, in situ, upper ocean crust^2,3^. Symbol colours indicate spreading rate as defined in ref. ^52^, with circles and squares representing drill sites located in the Atlantic and Pacific Oceans, respectively. Symbol size indicates basement penetration achieved at each site. Ridge flank carbon uptake is assumed to predominantly occur within the upper 300 m of the crust^2^, with greater penetration giving more confidence in the estimated carbon content of this sink. Error bars for the U1557 data are the standard deviation of the mean CO_2_ content of the cores, weighted by their drilled interval lengths (*n* = 17; Methods) and reflect the lithological heterogeneity of the talus breccia relative to typical upper crustal basalts. For other sites, data for each drillhole are reported with error bars representing reported propagated analytical errors^3^. Data and their sources are given in Extended Data Table 1.

We calculated the CO_2_ content of the U1557 talus breccia from the measured carbon concentrations of its carbonate-bearing components, their respective volume proportions of the recovered cores, and their densities (Supplementary Tables 1 and 2). The CO_2_ contents of individual cores range from 4.9 to 14.1 wt%, yielding an average bulk breccia deposit CO_2_ content of 7.5 ± 2.2 wt%, weighted by core interval lengths to normalize for variable core recovery (Methods and Supplementary Table 1). Previously cored upper ocean crust contains 0.2 to 4.2 wt% CO_2_, with Mesozoic crust typically having higher CO_2_ contents than Cenozoic crust and slow-spread crust containing more CO_2_ than faster-spread crust of similar age^2,3^ (Fig. 3 and Extended Data Table 1). The CO_2_ content of the U1557 breccia is therefore 2 to 40 times that of previously sampled upper ocean crust.

## A talus breccia-hosted global carbon cycle sink

The ridge flank hydrothermal carbon sink has previously been quantified from analyses of ~7 to 170-Ma upper ocean crust^2,3^. We consider the U1557 breccia-hosted carbonate to be a distinct additional ridge flank hydrothermal carbon sink because such talus breccias accumulate on top of the volcanic crust from which the global upper crustal carbon reservoir has previously been estimated, with the carbon they host therefore not included in those estimates. To represent an important reservoir in the long-term geological carbon cycle, talus breccia-hosted carbonate must occur elsewhere on the ridge flanks in substantial volumes. Given a talus breccia CO_2_ content 2 to 40 times that of upper crustal lavas, breccia deposits need only be 2.5 to 50% the thickness of the CO_2_-uptake zone in the underlying crust to host the same carbon sink. The ridge flank carbon sink has previously been estimated assuming uptake occurs predominantly within the upper 300 m of crust^2,3^, although only four of the studied drill holes penetrate to that depth (Fig. 3).

**Fig. 4:**
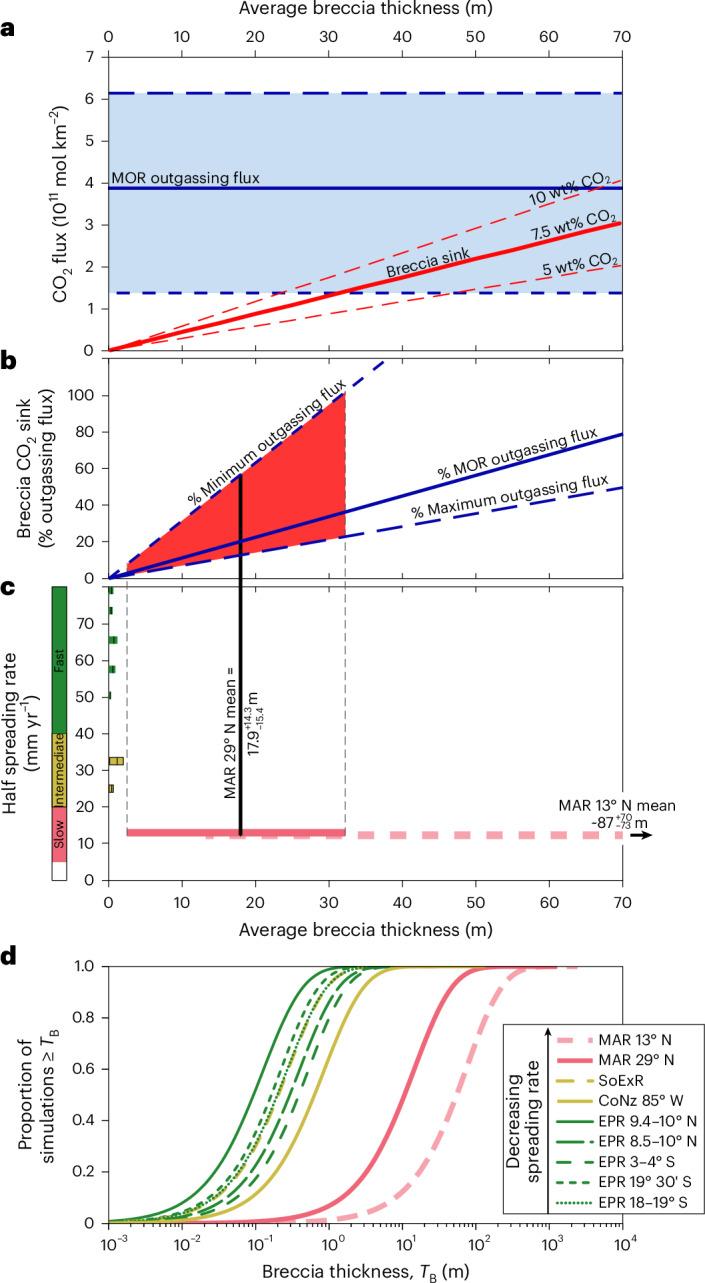
Comparison of MOR CO_2_-outgassing and talus breccia CO_2_-uptake fluxes. **a**, Comparison of the potential ocean crustal breccia CO_2_ sink with the estimated MOR outgassing flux^1^ (solid blue line) per km^2^ of ocean crust. The uncertainty in global MOR outgassing flux^1^ is indicated by the blue shaded region, with the maximum and minimum estimates indicated by long- and short-dashed blue lines, respectively. The magnitude of the talus breccia CO_2_ sink (red lines) depends on both its average thickness and CO_2_ content (7.5 wt% at Site U1557; solid red line). **b**, The CO_2_ sink hosted by talus breccia with 7.5 wt% CO_2_, expressed as a proportion of the estimated MOR CO_2_-outgassing flux to illustrate its relative magnitude, as a function of average breccia thicknesses. The solid, long- and short-dashed blue lines indicate the proportion of the mean, maximum and minimum estimates of the outgassing flux, respectively. **c**, Estimates of the average breccia thickness along segments of the slow-spreading Mid-Atlantic Ridge (MAR) (red), intermediate-spreading Southern Explorer (SoExR) and Cocos-Nazca (CoNz) Ridges (yellow) and fast-spreading East Pacific Rise (EPR) (green), calculated from observations of fault and talus geometries and tectonic strain using parametric bootstrapping (Methods and Supplementary Table 4). Coloured bars indicate uncertainty in the estimated mean thickness (1*σ* equivalent). **d**, Distributions of simulated breccia thicknesses (*T*_B_) used to calculate the mean thicknesses displayed in **c**. Breccia thickness in the MAR 13° N area (dashed light red bar/line in **c** and **d**, respectively) is only estimated on crust not affected by low-angle detachment faulting (that is, on the ridge flank opposite the oceanic core complexes) and is therefore interpreted with caution here (Methods). The red shaded region in **b** indicates the uncertainty in the extent to which talus breccia of the average thickness estimated for the MAR at 29° N (assuming a CO_2_ content of 7.5 wt%) could accommodate the CO_2_ outgassed during formation of the underlying crust, given the uncertainties in both the MOR CO_2_-outgassing flux and the estimated average breccia thickness.

Ocean crust is currently being produced at a rate of 3.4 km^2^ per year^33^, resulting in an estimated magmatic outgassing of 0.47–2.09 × 10^12^ moles CO_2_ (ref. ^1^)—equivalent to 14–61 × 10^10^ moles CO_2_ per km^2^ of new crust. The extent to which CaCO_3_ precipitation in talus breccias that subsequently accumulate on that crust buffers this release, globally, depends on both the bulk CO_2_ content and average thickness of the breccia, per km^2^ of crust (Fig. 4a).

**Extended Data Fig. 1:**
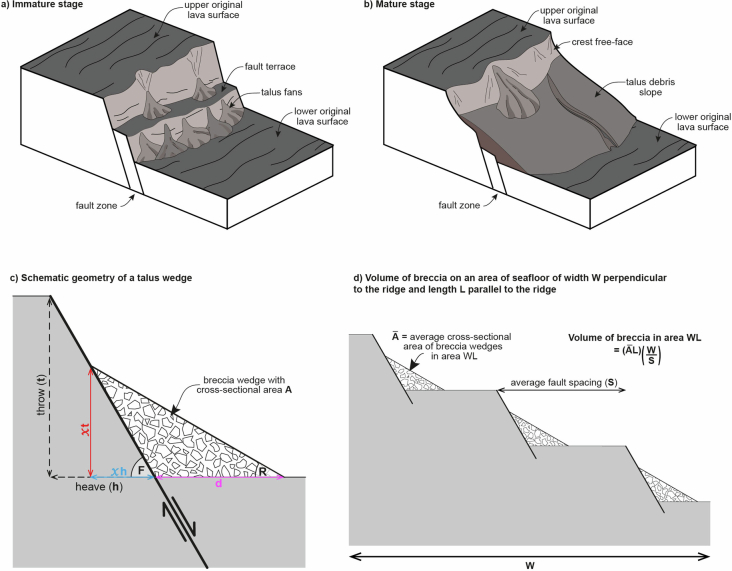
Talus breccia geometry. The geomorphological evolution of axial valley fault scarps and their talus deposits (**a**, **b**), based on deep-towed sidescan sonar images of the Mid-Atlantic Ridge between 24° 00’N and 24° 40’N (modified from^34^). **a**) Immature stage: small talus fans develop along fault terraces and the base of the fault scarp. **b**) Mature stage: a wedge of talus breccia covers the fault scarp, with its planar upper surface defined by the angle of repose of the breccia clasts. The exposed scarp (crest free-face) above the talus, is heavily eroded. **c**) The talus breccia deposits are modelled as triangular prisms parallel to the fault strike, with a triangular cross sectional area A. The fault has throw, heave, and dip angle of t, h, and F, respectively; the breccia deposit has a planar upper surface, defined by the angle of repose (R) of the clasts, and extends a distance d (pink arrow) from the fault scarp. If the proportion of the fault scarp covered by talus is given by x, then the height of the talus wedge is xt (red arrow) and the horizontal width of the slope scarp covered by talus is xh (blue arrow). **d**) the volume of breccia on an area of seafloor with width W perpendicular to the ridge, and length L parallel to the ridge axis, depends on the average cross-sectional area of the deposits ($\bar{A}$), their length (L), and the number of deposits in the area (W/S, where S is the average fault spacing).

At slow-spreading rates, talus breccia piles are common along the normal fault scarps of the modern axial valley walls, evolving from small talus fans to wedge-shaped deposits that cover most of the lower fault slope over ~1 Myr as the faults grow^34,35^ (Extended Data Fig. 1). The planar upper surface of mature deposits is controlled by the angle of repose of the talus clasts (typically 25–40° (ref. ^24^)). These breccia deposits are rafted across the ocean basin floor with the axial faults as spreading continues at the ridge, over which time their geometry may be modified.

To assess the average thickness of breccia potentially generated by axial valley faults we modelled their breccia deposits as ridge-parallel triangular prisms with their upper and lower surfaces defined by the angle of repose (*R*) and fault dip (*F*), respectively (Extended Data Fig. 1). The average thickness of breccia per km^2^ of ocean crust (*T*_B_), is given by:

1 $${T}_{{\rm{B}}}=\frac{{x}^{2}}{200}\dot{\varepsilon }\left[\frac{\tan F}{\tan R}-1\right]\bar{t}$$

where *x* is the proportion of the fault scarp covered by talus, $\bar{t}$ is the average fault throw and $\dot{\varepsilon }$ is the tectonic strain (%) across the plate boundary (Methods provide derivation).

**Extended Data Fig. 2:**
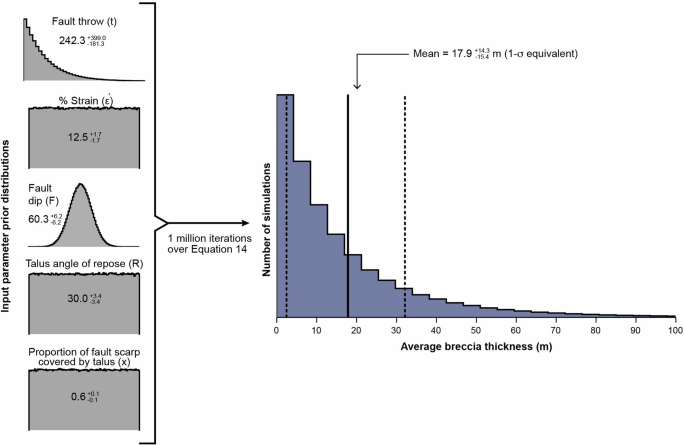
Breccia thickness model output. Breccia thickness model output for the Mid-Atlantic Ridge 29°N area, with robustly constrained uncertainty using parametric bootstrapping (Monte Carlo simulation; mean value with 1σ-equivalent errors shown by solid and dashed lines, respectively). Sampled prior probability distributions of the input parameters are shown to the left and listed in Supplementary Table 4.

Using detailed observations of normal fault and talus deposit geometries and tectonic strain along the modern Mid-Atlantic Ridge at 29° N (refs. ^21,35^) we estimate a mean talus breccia thickness of ${18}_{-15}^{+14}$ m (1*σ* equivalent) will accumulate on crust produced at this ridge segment, which has crustal architecture typical of slow-spread crust without low-angle oceanic detachment faults. The uncertainty in this estimate was robustly constrained using parametric bootstrapping (Methods and Extended Data Fig. 2). Assuming a CO_2_ content of 7.5 wt% and bulk density of 2,570 kg m^−^^3^, such an average talus breccia thickness would host ${7.8}_{-6.7}^{+6.3}$ × 10^10^ moles CO_2_ km^−^^2^. This is equivalent to ${20}_{-17}^{+16} \%$ of the best estimate of the CO_2_ released during formation of the underlying crust, although given the similar uncertainty in the MOR outgassing flux it could be equivalent to as much as 102% of the CO_2_ released (Fig. 4b).

**Extended Data Fig. 3:**
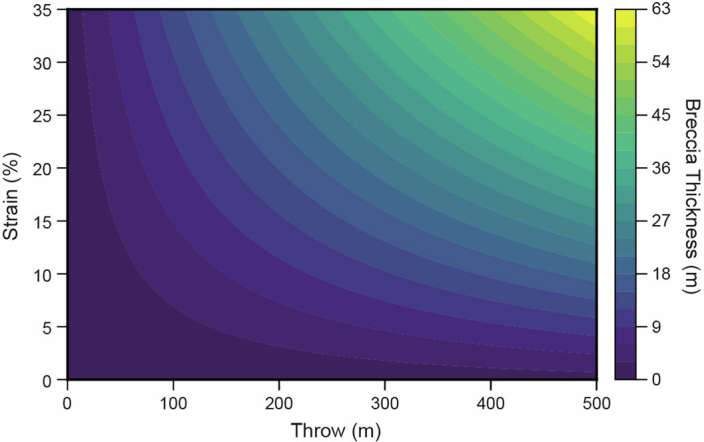
Contour plot showing the dependence of breccia thickness on strain (%) and average throw. Talus breccia thickness calculated using Eq. 14, assuming constant values for the proportion of the fault scarp covered by talus (x), its angle of repose (R), and fault dip (F), of 0.6, 30° and 60°, respectively.

**Extended Data Fig. 4:**
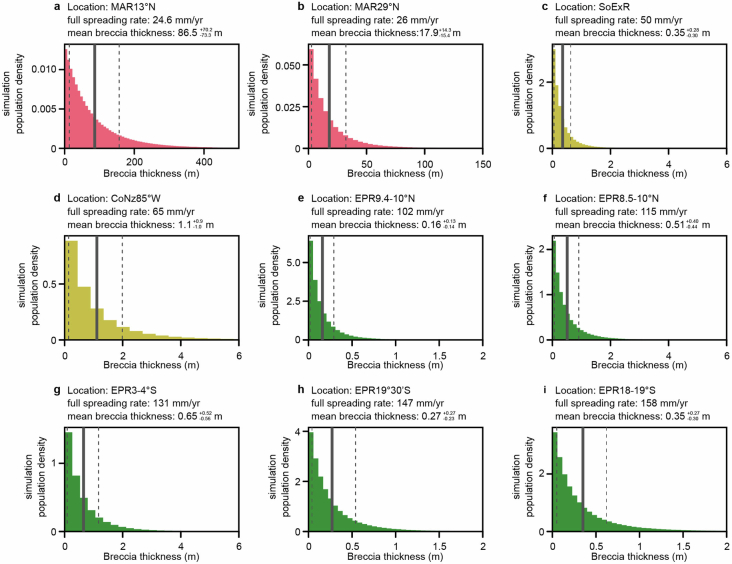
Breccia thickness model outputs for ridges spreading at different rates. Breccia thickness model outputs, with robustly constrained uncertainty using parametric bootstrapping (Monte Carlo simulation) for segments of the slow-spreading Mid-Atlantic Ridge (**a**,**b**; red), intermediate-spreading Southern Explorer (SoExR) and Cocos-Nazca (CoNz) Ridges (**c**,**d**; yellow) and fast-spreading East Pacific Rise (EPR) (**e**–**i**; green). Mean values with 1σ-equivalent errors shown by solid and dashed grey lines, respectively. The sampled prior probability distributions of the input parameters, and their sources, are listed in Supplementary Table 4. Within the MAR13°N area, at each latitude, we only include faults on the ridge flank without detachment faults.

Because average fault throws and tectonic strain vary substantially along the global MOR system, the average talus breccia thickness per km^2^ ocean crust is expected to vary substantially, too. Axial grabens are common on intermediate-spreading ridges, and faulting occurs at all spreading rates—albeit with smaller throws and lower tectonic strain at faster spreading rates^35^. Although the global average talus breccia thickness remains unknown, talus abundance is predicted to increase nonlinearly with decreasing spreading rate (Fig. 4d and Extended Data Figs. 3 and 4). Crucially, the global talus-hosted CO_2_ sink should therefore have varied in response to global changes in spreading rate^36^, in contrast to the CO_2_-outgassing flux, which is independent of spreading rate^1^.

## Prolonged CO_2_ uptake in talus breccias on slow-spread crust

**Fig. 5:**
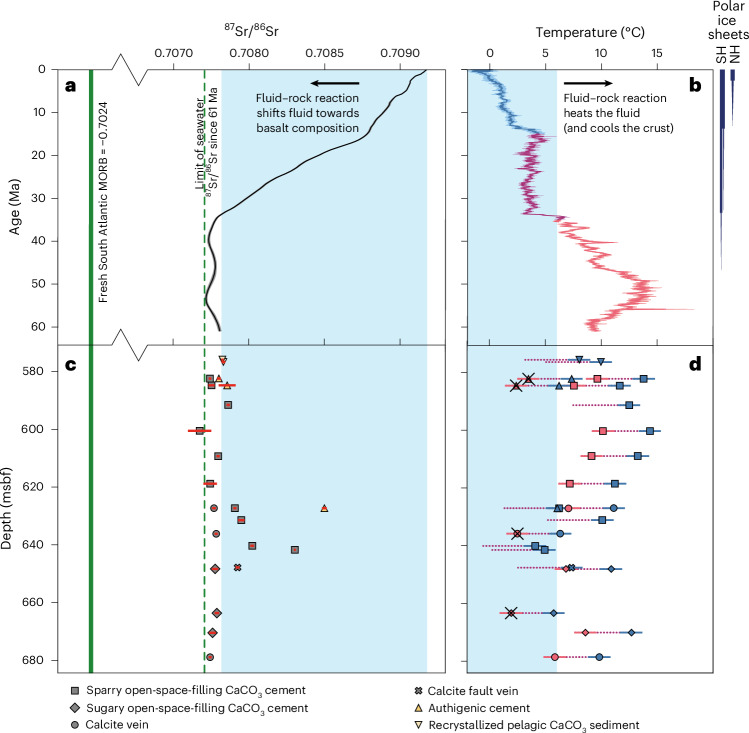
Comparison of fluid strontium isotopic composition and temperatures recorded by U1557 carbonates with seawater records since the crust formed. Symbol shapes indicate carbonate type. **a**, Seawater ^87^Sr/^86^Sr^40^; fluid–rock reactions shift fluid compositions towards fresh mid-ocean ridge basalt (MORB; solid green line^53^). **b**, Bottom-water temperature (Supplementary Methods). Polar ice sheet distributions to right, following ref. ^54^; SH, southern hemisphere; NH, northern hemisphere. **a**–**d**, Blue shading shows bottom seawater ^87^Sr/^86^Sr (**a**,**c**) and temperature ranges (**b**,**d**) since 34 Ma. **c**, Carbonate ^87^Sr/^86^Sr; data are presented as mean values and red error bars indicate analytical uncertainty (±2 SE; Supplementary Table 3). **d**, Precipitation temperatures calculated from carbonate δ^18^O following ref. ^55^, assuming basement fluids have δ^18^O of contemporaneous seawater^42,43^. As U1557 carbonates could have precipitated at any time since 61 Ma, endmember temperatures are calculated for ice-free oceans (δ^18^O_seawater_ = −1‰; red symbols) and oceans with established ice sheets (δ^18^O_seawater_ = 0‰; blue symbols), with intermediate values (purple dashes) when ice sheets wax/wane (corresponding to red, blue and purple intervals in **b**, respectively). Data are presented as calculated temperatures with conservative error bars of ±1 °C reflecting the potential uncertainty due to the choice of thermometer (Supplementary Methods for discussion). Where ^87^Sr/^86^Sr compositions require formation since 34 Ma (blue shaded region of **c**) ice-free temperatures are not shown. Precipitation temperatures colder than the overlying oceans are not possible (‘ice-free’ temperatures within blue shaded region of **d**), as indicated by crosses.

CaCO_3_ precipitated within ocean crust records the Sr isotopic composition and temperature of the fluids it forms from^37^. Strontium exchange during fluid–rock reaction lowers the ^87^Sr/^86^Sr ratios of ridge flank basement fluids from their initial seawater compositions towards that of basalt (~0.703^38^), with most fluid heated as it reacts with, and cools, the crust^37,39^. The ^87^Sr/^86^Sr ratio of seawater since 61 Ma is well established from marine biogenic carbonate sediments^40^ and has ranged between a minimum of 0.707715 ± 0.000010 (53.5 million years ago) and the modern ratio of 0.70918 (Fig. 5a). Whether the U1557 carbonates record seawater Sr isotopic compositions or contain a basaltic Sr component depends on when they precipitated. Approximately half of the sparry carbonate cements have Sr isotopic compositions between 0.707865 and 0.708305 (Fig. 5c and Supplementary Table 3), falling within the blue shaded region of Fig. 5a, consistent with their formation either from seawater between 34 and 23 Ma (that is, 27 to 38 Myr after the crust formed) or more recently if they precipitated from a fluid containing some basalt-derived Sr. Most of these carbonates formed between 630 and 650 metres below seafloor (mbsf) in an interval overlying a carbonate-cemented fault zone that may have promoted prolonged fluid flow through the breccia deposit (Fig. 2c). However, the remaining vein and cement carbonates have ^87^Sr/^86^Sr ratios lower than 34 Ma seawater (Fig. 5c) and could have precipitated as early as 0–5 Myr after crustal formation, given the fluctuations in seawater composition between 61 and 34 Ma (Fig. 5a).

Precipitation temperatures determined from carbonate oxygen isotopic compositions (δ^18^O; Supplementary Table 3) provide additional constraints on when the carbonates precipitated and the nature of the fluid they formed from. Calculated temperatures depend on the fluid δ^18^O, which is assumed to remain unmodified during low-temperature reactions with basalt^37,39,41^. Between 61 and 35 Ma, the oceans were ice free, with seawater δ^18^O of −1‰ (refs. ^42,43^). Using this ice-free fluid composition yields temperatures colder than 61–35 Ma seawater for many of our samples (Fig. 5b,d). However, the upper crust, and hence fluids flowing through it, cannot be cooler than bottom seawater. This suggests these carbonates formed more recently when the oceans were cooler (Fig. 5b) from fluids warmer than contemporaneous seawater, with a basaltic Sr component. The minimum duration of carbon uptake is recorded by an authigenic cement overgrowing and hence post-dating void-filling carbonate. It has a more radiogenic Sr isotopic composition of 0.7085, the composition of 19-Ma seawater, requiring precipitation at least 42 Myr after the crust formed (Fig. 5a,c).

Collectively, the carbonate cements, veins and matrix from the U1557 talus breccia record circulation of <15 °C seawater and/or seawater-derived fluids modified by reaction with basalt through the breccia for at least 42 million years on the southern Mid-Atlantic Ridge flank. This is substantially longer than observations from well-sedimented ridge flanks with more subdued basement topography^44^. This suggests that spreading rate not only influences the extent of CO_2_ uptake by ocean crust but also the duration of basement carbonate precipitation.

## Spreading rate and crustal age controls on carbon uptake

The discovery of a talus breccia-hosted carbon reservoir on the western flank of the slow-spreading southern Mid-Atlantic Ridge highlights the importance of upper crustal architecture when modelling the role of mid-ocean spreading in the long-term geological carbon cycle. The high primary porosity and large surface area of the U1557 basaltic talus breccia allow it to act as a geological sponge for the precipitation of dissolved seawater carbon in carbonate minerals. Other types of porous basaltic breccia form along the ridge axes through both magmatic processes and the collapse of volcanic cones^45^ or through mass wasting along oceanic fracture zones^46^ or on the flanks of seamounts. We would expect CaCO_3_ to precipitate in these breccias as well, as their host crust ages. U1557 cored the base of a faulted basin interpreted to be typical of axial valley normal faulting, indicating that CaCO_3_-cemented talus breccias are probably a global phenomenon on slow- to intermediate-spreading-rate crust. Consequently, past variations in the global lengths of slow-, intermediate- and fast-spreading ridges^36^ probably caused shifts in the relative magnitude of the carbon fluxes during mid-ocean ridge CO_2_ outgassing and carbonate formation on the ageing ridge flanks. The tectonic component of plate divergence at slow-spreading ridges that generates talus breccias also results in rough basement topography that remains incompletely covered by sediments for tens of millions of years on the ridge flanks, promoting prolonged fluid flow and carbonate precipitation in the upper crust. As spreading rate also influences the duration of CO_2_ uptake, models of the past contributions of ridge flanks to the long-term carbon cycle need to also allow for variations in the age-area distribution of the ocean crust^36^ as a function of spreading rate.

## Methods

The magnitude of the carbon sink that talus breccias accumulated on top of the ocean crust constitute depends on the breccia’s bulk CO_2_ content and its average thickness, per km^2^ of crust.

### Quantifying the carbon content of the U1557 talus breccia

To quantify the talus breccia-hosted carbon sink it is necessary to know the carbon content of the carbonate-bearing components in the breccia. On the basis of examination in hand specimen and thin sections and the results of shipboard X-ray diffraction analyses^17^, sparry and sugary carbonate cements constitute pure or almost pure calcite, which contains 44 wt% CO_2_. The mean CO_2_ content of the recrystallized pelagic carbonate sediment matrix present in the uppermost ~20 m of the breccia (43.3 wt%, *n* = 5; Supplementary Table 2) indicates that it is near-pure carbonate, consistent with the composition of the overlying sedimentary sequence^17^. In contrast, the microcrystalline authigenic cement from below ~20 m sub-basement constitutes a mixture of phillipsite zeolite and carbonate material. This material was estimated to constitute approximately 50 vol% carbonate based on petrographic observations^17^. Three samples of the authigenic material have CO_2_ contents ranging from 17 to 28 wt% (Supplementary Table 2), with a mean value of 26 wt%, consistent with the petrographic observations. Carbonate also replaces primary minerals and fills interstitial space in the basalt clasts, which consequently have CO_2_ contents ranging from 0.1 to 5.1 wt% with a mean of 1.0 wt% (*n* = 46; Supplementary Table 2).

The CO_2_ content of each core, [CO_2_]_*x*_ (Supplementary Table 1), was calculated from the volume proportions of the CO_2_-bearing components in each core (*V*_*i*__−__*x*_) following:

2 $${\left[{\mathrm{CO}}_{2}\right]}_{x}=\mathop{\sum }\limits_{i}{\left[{\mathrm{CO}}_{2}\right]}_{i-x}=\mathop{\sum }\limits_{i}{V}_{i-x}\left(\frac{{\rho }_{i}}{{\rho }_{x}}\right){\left[{\mathrm{CO}}_{2}\right]}_{i}$$

where [CO_2_]_*i*__−__*x*_ is the contribution to [CO_2_]_*x*_ from component *i*, ${\rho }_{x}$ is the bulk density of core *x*, and ${\rho }_{i}$ and [CO_2_]_*i*_ are the density and CO_2_ content (wt%; Supplementary Table 1) of component *i*, respectively. The bulk density of each core was calculated from the mean bulk densities for Site U1557 breccia clasts, sediment matrix and authigenic cement determined during shipboard physical properties measurements (2,610; 2,400 and 2,300 kg m^−^^3^, respectively^17^), assuming the mean density of non-carbonate secondary minerals is that of breccia clasts and the density of calcite cement is 2,710 kg m^−^^3^:

3 $${\rho }_{x}=\mathop{\sum }\limits_{i}{V}_{i-x}{\rho }_{i}$$

The average CO_2_ content of the breccia deposit was calculated from core CO_2_ contents, weighted by core interval lengths (*L*_*x*_) to normalize for variable core recovery:

4 $${\left[{\mathrm{CO}}_{2}\right]}_{\mathrm{Breccia}}=\frac{\mathop{\sum }\limits_{x}{L}_{x}{\rho }_{x}{\left[{\mathrm{CO}}_{2}\right]}_{x}}{\mathop{\sum }\limits_{x}{L}_{x}{\rho }_{x}}$$

where ${\sum }_{x}{L}_{x}{\rho }_{x}$ is the bulk density of the breccia (2,570 kg m^−^^3^).

### Thickness of talus breccia accumulated on ocean crust

To assess the potential global volume of talus breccia on slow-spread crust, we model talus breccia deposits as ridge-parallel triangular prisms with their upper and lower surfaces defined by the angle of repose (*R*) and fault dip (*F*), respectively (Extended Data Fig. 1). The triangular cross-sectional area of the breccia wedge (*A*) is given by:

5 $$A=\frac{1}{2}\left({xt}\right)\left(d+{xh}\right)-\frac{1}{2}\left({xt}\right)\left({xh}\right)=\frac{1}{2}{xtd}$$

where *t* and *h* are the fault’s throw and heave, respectively; *x* is the proportion of the fault scarp covered by talus, such that *xt* is the height of the breccia pile and *xh* is the horizontal width of fault covered by breccia; and *d* is the distance the talus pile extends from the fault (Extended Data Fig. 1). From trigonometry:

6 $$\tan R=\frac{{xt}}{d+{xh}}$$

and

7 $$\tan F=\frac{t}{h}$$

Combining equations (6) and (7) gives the expression:

8 $$d={xt}\frac{\left(1-\frac{\tan R}{\tan F}\right)}{\tan R}$$

Consequently, the cross-sectional area of the breccia wedge can be expressed in terms of *x*, *t*, *R* and *F*:

9 $$A=\frac{{x}^{2}{t}^{2}}{2\tan R}\left[1-\frac{\tan R}{\tan F}\right]$$

The volume of breccia, *V*_B_, on an area of seafloor of width *W* perpendicular to the ridge axis and length *L* parallel to the ridge is the product of the average volume of breccia wedges in the area ($\bar{A}L$, where $\bar{A}$ is the average cross-sectional area of the wedges) and the number of breccia wedges across that crustal width (*W*/*S*, where *S* is the average fault spacing; Extended Data Fig. 1d):

10 $${V}_{{\rm{B}}}=\left(\bar{A}L\right)\left(\frac{W}{S}\right)=\frac{{x}^{2}{\left(\bar{t}\right)}^{2}}{2\tan R}\left[1-\frac{\tan R}{\tan F}\right]L\frac{W}{S}$$

where $\bar{t}$ is the average fault throw. The average thickness of breccia per unit area of ocean crust, *T*_B_ (the thickness if the wedge-shaped deposits were spread out to form a layer of uniform thickness, evenly across the entire area), is therefore given by:

11 $${T}_{{\rm{B}}}=\frac{{V}_{{\rm{B}}}}{{WL}}=\frac{{x}^{2}{\left(\bar{t}\right)}^{2}}{2S\tan R}\left[1-\frac{\tan R}{\tan F}\right]$$

The proportion of plate divergence accommodated by faulting (% strain; $\dot{\varepsilon }$) increases with decreasing spreading rate^35^. The strain across the plate boundary is a function of fault size and spacing:

12 $$\dot{\varepsilon }=100\left(\frac{h}{S}\right)$$

Combining equations (7) and (12), the fault spacing can be expressed as:

13 $$S=100\left(\frac{\bar{t}}{\dot{\varepsilon }\tan F}\right)$$

and combining equations (11) and (13), the average breccia thickness per unit area of ocean crust is given by:

14 $$\begin{array}{rcl}{T}_{{\rm{B}}} & = & \frac{{x}^{2}}{200}\dot{\varepsilon }\left[\frac{\tan F}{\tan R}\right]\left[1-\frac{\tan R}{\tan F}\right]\bar{t}\\ & = & \frac{{x}^{2}}{200}\dot{\varepsilon }\left[\frac{\tan F}{\tan R}-1\right]\bar{t}\end{array}$$

The average breccia thickness is therefore proportional to both fault throw and strain (Extended Data Fig. 3). Normal faulting occurs across the full spectrum of mid-ocean spreading rates. Along ridges spreading at full rates below 60–70 mm yr^−1^ sub-axial magma chambers are episodic, and the melt supply is <90% of that required by plate separation^35^. Tectonic strain accommodates the remainder of the plate separation, with the average fault throw orders of magnitude greater than at fast-spreading ridges (Supplementary Table 4). As both average fault throw and strain increase with decreasing spreading rate, the average talus breccia thickness is expected to be greatest on slow-spread crust. However, breccia should accumulate to some extent on crust produced at all spreading rates if there is a component of tectonic strain across the plate boundary.

At slow-spreading rates, the proportion of plate separation accommodated by tectonic strain varies considerably, depending on the mode of crustal accretion. Along some ridge segments very large-offset (up to 10 km (ref. ^56^)) low-angle normal faults, known as detachment faults and with their uplifted footwalls termed oceanic core complexes, typically accommodate 40 to 80% of plate separation^22^ and up to 100% at ultraslow spreading rates^57^. Between 12° 30’ N and 35° N on the Mid-Atlantic Ridge, it is estimated that detachment faults occur along at least 50% of the ridge axis, where a single fault accommodates ~50% of plate separation^58^. Although detachment fault surfaces are observed to be draped in talus breccias^56^ and/or to produce ultramafic mass-wasting deposits^24^, these deposits have different geometries to that depicted in Extended Data Fig. 1 and we therefore do not model their thickness using equation (14) here. To fully quantify the global ocean crustal talus breccia abundance, the geometries of the talus deposits associated with detachment faults need to be further investigated.

Along more magmatically robust slow-spreading ridge segments, fault size (that is, heave and throw) and spacing are highly variable; at spreading segment centres, faults typically have moderate offsets, whereas at spreading segment ends, the faults have greater offsets but are more widely spaced^21^. Given this inverse relationship between heave and fault spacing, the resultant total tectonic strain does not vary substantially along axis of magmatic segments of slow-spreading ridges^21^.

To explore how the variability in the geometry of non-detachment normal faults along mid-ocean ridges may impact talus breccia generation we model breccia thickness (*T*_B_) at ridge segments spreading at slow to fast rates. Here we apply a parametric bootstrapping (Monte Carlo simulation) technique to robustly constrain the posterior probability distribution of breccia thickness at each location. We apply prior probability distributions of the discrete parameters in equation (14). To capture the variability in fault geometries at each location modelled, this requires detailed observations of the fault populations’ geometries. Unfortunately, the availability of such datasets is limited.

Here we consider observations along the entire slow spreading (~26 mm yr^−1^ full rate) segment of the Mid-Atlantic Ridge (MAR) at 29° N (ref. ^21^) as representative of typical axial valley morphology of slow-spreading ridges that generate non-detachment-fault seafloor. Deep-towed sidescan sonar images along this ridge segment were collected with an ~10-m spatial resolution along 0.5-km spaced east–west transects across the NNW- (010) trending ridge axis, to constrain the geometry of faulting along the ridge segment^21^. The mean heave of all faults identified was reported to be 197 ± 293 m (1-sigma). Furthermore, for faults with *h* < 500 m, the cumulative heave-frequency distribution was found to be characterized by an exponential distribution^21^. Assuming a theoretical normal fault dip of 60° (refs. ^59–61^), the mean observed heave equates to mean throw of ~350 m (equation (7)). To model the average breccia thickness expected to accumulate on crust generated along this segment, fault throw is therefore designated as an exponential distribution with a mean value of 350 m, maximum of 1,700 m and minimum of 1 m based on the observed distribution of fault throws^21^. Strain is designated as a uniform distribution ranging between 10 and 15% (ref. ^21^). Fault dip is designated as a Gaussian distribution with a median value of 60°, a maximum dip angle of 75° and minimum dip angle of 45° based on theoretical fault dips and observed fault scarp geometries^62^. The angle of repose is designated as a uniform distribution ranging between 25 and 35°, based on slopes determined from high-resolution bathymetry of basaltic debris deposits at 16° 38’ N on the Mid-Atlantic Ridge that were consistent with the angle of repose in non-cohesive granular flow experiments^24^. The proportion of the fault scarp covered by talus is designated as 0.6 ± 0.1, based on observations of the proportion of the axial valley wall covered by talus deposits at 16° 38’ N on the Mid-Atlantic Ridge^24^. These prior distributions were randomly sampled and iterated 1,000,000 times over equation (14) to estimate a mean breccia thickness of 10 m, with a robustly estimated uncertainty of 8.4 m (1-*σ* equivalent; Extended Data Fig. 2).

To investigate the impact that the proportion of plate separation accommodated by tectonic strain has on fault geometry, and hence breccia accumulation, of non-detachment faults along slow-spreading ridges, we use the same approach to model the mean breccia thickness expected to accumulate on crust accreted along the MAR near 13° N. We use the results from near-bottom sidescan sonar/bathymetry profiler surveys of fault geometries along 24 profiles across two Mid-Atlantic Ridge segments between 13° 14’ N and 13° 54’ N (ref. ^56^), along which a greater proportion of plate separation is accommodated by tectonic strain than at 29° N, and there are several detachment faults despite a similar full-spreading rate (24.6 mm yr^−1^). The cumulative tectonic strain within the past 1.86 Myr ranges from 25 to 30% between the detachment faults and increases to <42% at the latitudes of the detachment faults^56^. Here we only model breccia accumulation on the ridge flank without detachment faults at each latitude (that is, the west flank of the MAR to the north of 13° 38’ N and the east flank to the south^56^). These non-detachment faults have current average heave and throw of 820 m and 177 m, respectively^56^. However, the tectonic component of extension in this region resulted in significant crustal tilting^56^, and their original dip of 65 ± 12° (ref. ^56^) equates to a mean pre-rotation throw of ~600 m. Given that both the strain and fault throw are higher along the MAR at 13° N than at 29° N, a greater average thickness of talus breccia is expected (Extended Data Fig. 3). Assuming the same values for the angle of repose and proportion of the fault scarp covered by talus as used to model the MAR at 29° N, our parametric bootstrapping approach yields an estimated mean breccia thickness of $87{\pm }_{73}^{70}$ m (Supplementary Table 4 and Extended Data Fig. 4). This is five times thicker than our estimate of the mean breccia thickness along the MAR at 29° N, with such a thickness of breccia potentially capable of providing a sink for the full MOR outgassing flux (Fig. 4). However, we interpret this result with caution because (1) we only model breccia accumulation on the portion of non-detachment crust in this area and (2) the scale of faulting here is greater than in the area in which observations of talus geometries (angle of repose, *R*, and the proportion of fault scarp covered, *x*) are reported. Nonetheless, these results highlight the need to better constrain the variation in fault geometries, strain and the proportion of fault scarps covered by talus along slow-spreading ridges to more robustly quantify the global abundance of talus breccia on slow-spread crust.

Following the same approach, we also model the mean breccia thickness expected to accumulate on crust produced at faster-spreading ridge segments, given their observed fault population geometries and estimates of the proportion of plate separation accommodated across them by tectonic strain (Supplementary Table 4 and Extended Data Fig. 4). Although there is considerable uncertainty in our estimates of the mean breccia thickness at different ridge segments, and it is probably variable even between segments spreading at similar rates, the stark contrasts in tectonic strain and fault scale between slow- and fast-spreading ridges indicate that the volume of talus breccia they host also differs by several orders of magnitude.

### Global abyssal hill mass-wasting fluxes and breccia thickness

Given the spatial variability in breccia abundance and uncertainties in our approach, we do not attempt to extrapolate our results to calculate the global average breccia thickness on ocean crust. However, here we compare the range of our average breccia thickness estimates at different spreading rates to the global average breccia thickness that might be expected based on independent estimates of the global mass-wasting flux on fault-bounded abyssal hills^63^. Hughes et al.^63^ use a nonlinear topographic diffusion model, constrained with global observations of abyssal hill topography, to estimate an abyssal hill mass-wasting flux of 24–1,428 × 10^6^ m^3^ yr^−1^, assuming faults dip 45°, the critical angle above which erosion occurs is 40° and volume is conserved (that is, porosity of talus = porosity of fractured bedrock). Given the current ocean crustal production rate of 3.4 km^2^ per year (ref. ^33^), this would equate to a global average breccia thickness of 7–420 m. However, the calculated primary porosity of the U1557 talus deposit (~20%; Fig. 2) indicates that volume is not conserved and hence the estimated global mass-wasting flux would equate to a global average breccia thickness between 9 and 525 m. However, Hughes et al.^33^ calculate the global abyssal hill mass-wasting flux (*Q*_global_; m^3^ yr^−1^) following:

15 $${Q}_{\mathrm{global}}=\frac{{A}_{\mathrm{ave}}}{\tau }{LN}$$

where *A*_ave_ is the cross-sectional area (ridge perpendicular; 4,978–22,207 m^2^) eroded from an average height (200 m) abyssal hill, *τ* (in years) is the time for the hill to form, *L* is the global MOR length (60,000 to 70,000 km) and *N* is the number of actively growing hills in a 30-km-wide active fault window either side of the MOR. *τ* is calculated to be ~20–200 ka, given the abyssal hill height and estimated vertical fault slip rate (0.72–9.25 mm yr^−1^) and, crucially, assuming only one fault is active at a time. On the basis of their observation that the average abyssal hill spacing, *S*, is 3 km, Hughes et al.^33^ determine that the number of active faults at any time, *N*, is 20. However, this is inconsistent with their assumption that only one fault is active, when determining *τ*. If more than one fault is active at a time (that is, the strain is distributed across multiple faults), it must take longer to grow each hill. Given full-spreading rates of 30, 60 and 100 mm yr^−1^ the 60-km-wide active fault window represents 2, 1 and 0.6 Myr of crustal accretion, respectively. Consequently, at these spreading rates, abyssal hill bounding faults should only be active for between 1 and 46% of the time they spend traversing the active fault window as the crust spreads away from the MOR. This suggests that Hughes et al.^33^ have over-estimated the global abyssal hill mass-wasting flux by a factor of between 2 and 100, depending on spreading rate, and that the average breccia thickness calculated above based on these fluxes is similarly over-estimated. Furthermore, if one is interested in the time-averaged global abyssal hill mass-wasting flux, then it is the volume of talus produced by a pair of faults (growing concurrently either side of the MOR) and the time taken to produce the crust between them and the next fault pair (dependent on fault spacing and spreading rate) that are important, rather than the time taken to grow each pair of hills:

16 $$\begin{array}{ll}{Q}_{\mathrm{global}}({{\rm{m}}}^{3}\,{\mathrm{yr}}^{-1})\\ =\displaystyle\frac{2[{\mathrm{volume}}\,{\mathrm{of}}\,{\mathrm{single}}\,{\mathrm{talus}}\,{\mathrm{wedge}}\,{\mathrm{of}}\,{\mathrm{length}}\,L({{\rm{m}}}^{3})]}{[{\mathrm{spreading}}\,{\mathrm{interval}}\,({\mathrm{years}}\,){\mathrm{between}}\,{\mathrm{faults}}\,{\mathrm{with}}\,{\mathrm{spacing}}\,S]}\\ =2[{A}_{\mathrm{ave}}L]/\left[\displaystyle\frac{S}{{\mathrm{half}}\,{\mathrm{spreading}}\,{\mathrm{rate}}}\right]\end{array}$$

Assuming the global abyssal hill range comprises 3-km-spaced hills that are 200-m high (that is, ignoring the observed variations in abyssal hill geometry with spreading rate), *A*_ave_/*S* yields a more reliable estimate of average breccia thickness, on the order of 2 to 9 m. This range based on the cross-sectional area of eroded material modelled by Hughes et al.^33^ is in good agreement with our estimates of average breccia thickness on slow- to intermediate-spreading-rate crust with fault scarps of similar average relief (Supplementary Table 4 and Extended Data Fig. 4).

## Online content

Any methods, additional references, Nature Portfolio reporting summaries, source data, extended data, supplementary information, acknowledgements, peer review information; details of author contributions and competing interests; and statements of data and code availability are available at 10.1038/s41561-025-01839-5.

## Supplementary information

Supplementary Information Supplementary Methods, Fig. 1, captions for Tables 1–6 and list of South Atlantic Transect IODP Expedition 390 and 393 scientists.

Supplementary Tables Supplementary Tables 1–6.

## Figures and Tables

**Extended Data Table 1:**
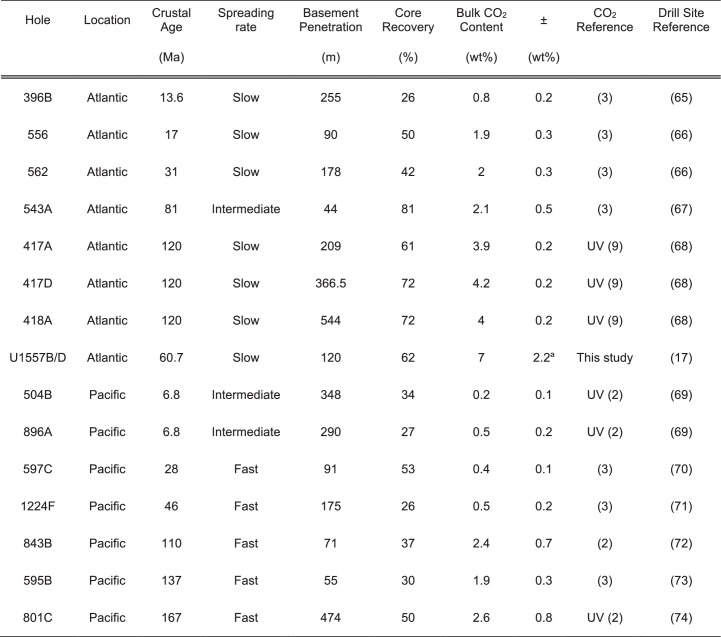
Estimated bulk CO_2_ contents of upper crustal sections sampled by scientific ocean drilling

Drill Site data from refs. ^65–74^.

^a^Uncertainty is the standard deviation of the mean CO_2_ content of the U1557 breccia cores, weighted by their drilled interval lengths (Methods and Supplementary Table 1). See details in CO_2_ references for methods used to estimate uncertainty at other sites. UV = Upper Volcanics bulk CO_2_ content reported.

## Data Availability

All data related to this manuscript can be found in Extended Data Figs. 1–4, Extended Data Table 1 and Supplementary Tables 1–6 and are also available along with the source data via Zenodo at 10.5281/zenodo.16794553 (ref. ^64^).

## Code availability

Our code is available from the corresponding author on reasonable request.
