# Supplementary material for: A geological carbon cycle sink hosted by ocean crust talus breccias
Source: Nat Geosci. 2025 Nov 24;18(12):1279–86. doi: 10.1038/s41561-025-01839-5 (PMC12685744; doi:10.1038/s41561-025-01839-5)
Supplement: Supplementary file 1 — Supplementary Methods, Fig. 1, captions for Tables 1–6 and list of South Atlantic Transect IODP Expedition 390 and 393 scientists. [file 41561_2025_1839_MOESM1_ESM.pdf]

nature geoscience

Supplementary information https://doi.org/10.1038/s41561-025-01839-5

A geological carbon cycle sink hosted by ocean crust talus breccias

In the format provided by the authors and unedited

SUPPLEMENTARY INFORMATION:

Contents:

Supplementary Methods: page 2

Site Description and Core-Logging Methods page 2

Geochemical Analytical Methods page 4

Carbonate Precipitation Temperature Calculations page 7

Deep Ocean Temperature Record page 9

Supplementary Figures:

Supplementary Figure 1. Site U1557 core-logging results and CO_2 content. page 10

Supplementary Tables provided in additional files:

Supplementary Table 1. Site U1557 core-logging results and CO_2 content. page 10

Supplementary Table 2. CO_2 concentrations of U1557 whole rock samples. page 11

Supplementary Table 3. Stable (C, O) and radiogenic (Sr) isotope compositions of carbonate samples. page 11

Supplementary Table 4. Breccia thickness model parameters and results. page 11

Supplementary Table 5. Validation of logged carbonate abundances. page 12

Supplementary Table 6. Comparison of inorganic carbonate oxygen isotope thermometers. page 12

Supplementary References. page 14

List of South Atlantic Transect IODP Expedition 390 & 393 Scientists. page 19

Supplementary Methods

Site Description and Core-logging Methods

Cores analyzed in this study were recovered from International Ocean Discovery Program (IODP) South Atlantic Transect Holes U1557B and U1557D during IODP Expeditions 390C and 390[1]. Site U1557 is in the western South Atlantic Ocean ~1250 km west of the Mid-Atlantic Ridge (MAR) at a water depth of ~5000 m. It is located on 60.7 Ma ocean crust formed at a slow spreading rate (~13.5 mm/yr half-rate) at the MAR. Site U1557 is located toward the eastern edge of a ~17.5 km wide basin that is filled with sediments to a uniform bathymetric level. The basin sits between two approximately north-south trending, strongly faulted basement ridges, which have steep flanks and very thin or no sediment cover. Basement topography changes considerably within this basin resulting in large differences in sedimentary cover. Site U1557 is located at the deepest point of a 2 km wide subbasin, where the sediment is 564 m thick. Hole U1557B was cored to a total depth of 574 meters below seafloor (mbsf), penetrating the entire sediment succession and ~10 m into basement with excellent preservation of the sediment-basement interface. A reentry system with casing extending ~5 m into basement was installed in Hole U1557D, with coring beginning ~10 m below the sediment-basement interface and extending ~120 m into the basement. The sediment sequence comprises ~290 m of Eocene to recent alternating silty clay and nannofossil ooze layers, overlying ~275 m of Paleocene to Eocene nannofossil/calcareous chalk. The ~120 m composite basement section cored in Holes U1557B and U1557D, with an average core recovery of 63%, comprises talus breccias that contain a range of basaltic clast types derived predominantly from pillow lavas. Ubiquitous volcanic glass clasts are almost all completely altered with rare fresh glass.

The amount of carbonate present in the basement cores from Holes U1557B and U1557D was quantitatively logged on a per-core-piece basis using a combination of visual estimation and ground truthing to grey-scale digital images. The volume proportions of open space-filling carbonate cement, recrystallized pelagic sediment matrix, authigenic cement and macroscopic open porosity were determined (together with the proportions of other secondary minerals and of the bulk alteration of igneous lithologies; not presented here) by visual core logging of the archive half of the cores aboard the drilling vessel *JOIDES Resolution*. Carbonate cement abundances were estimated individually for each recovered piece of core (typically 1-50 cm in length) or for two or more intervals within a piece where there was a sharp discontinuity in cement abundance within a single piece. It was assumed that the area proportion of a given logged feature on the split core surface was proportional to the volume proportion (vol%) of that feature. Hydrothermal veins were also logged, with their depth in the hole, width, length, and mineralogy recorded. The volume of each core comprising calcium carbonate veins was calculated and included in the estimates of total carbonate abundance. All alteration logging data (including pelagic sediment matrix and authigenic cement) were normalized to take account of the proportions of the core composed of vein and cement filling minerals such that the total of all logged components sum to 100 vol%.

The accuracy of the proportions of carbonate cement determined by visual core logging was verified using scanned images of the core surface and the image processing and analysis tool ImageJ. Thirteen core sections representative of the range of carbonate cement abundance (0.93–21.5 vol%) were chosen. For each, a photographic scan of the core section was converted to grayscale and the area of the image constituting rock or carbonate was determined by manually setting a brightness threshold in ImageJ. Since the rock clasts and carbonate cement each have

markedly different brightness this resulted in good delineation of the different components. Image analysis results are positively correlated with those from visual core logging, with an R^2 value of 0.78 (Supplementary Fig. 1). The data lie close to a 1:1 line and a best fit linear regression through the origin has a slope of 0.95. This regression analysis indicates that the data from core logging show a good degree of accuracy and do not show any significant systematic bias; the root mean square misfit of the logging data is 2.37 vol%, mean relative misfit is 24% and the mean raw misfit is 0.09 vol%, close to zero (Supplementary Table 5). Average abundances of each carbonate-bearing component (open space-filling cement, pelagic sediment matrix, authigenic cement) and open porosity were calculated on a core-by-core basis and reported as their mean vol% of the rock recovered in each core interval (Supplementary Table 1).

Geochemical Analytical Methods

Whole rock samples were prepared for carbon content analyses at the National Oceanography Centre Southampton (NOCS), University of Southampton. Core samples were cut to yield representative samples of the various carbonate-bearing breccia components (basalt clasts, recrystallized pelagic sediment, and authigenic carbonate + zeolite cement) through Hole U1557D, using a diamond saw. Cut surfaces were ground to remove contamination from the saw blade. Samples were then washed and ultra-sonicated in Milli-Q 18.2 MΩ cm^{-1} water and left to dry overnight in an oven at 65°C. After drying, samples were crushed using a manual iron fly-press with samples wrapped in paper between plastic chopping boards to reduce potential contamination. Samples were then powdered using a chrome-steel jar mill and puck in a Rocklabs mill.

Carbon concentrations were determined using a LECO Carbon and Sulphur (CS744) analyzer (Michigan, USA) at NOCS. This instrument ignites powdered samples and converts carbon to CO_2 using O_2 gas to determine CO_2 concentrations using an infrared detector. Powdered samples of 0.1 ± 0.02 g were weighed and loaded into crucibles with ~1 g of LECO iron filings and ~1 g of LECOCEL tungsten and tin accelerants added. The instrument was calibrated using LECO certified carbon standards: 502-928; 502-909; 502-894; 502-698; and calcium carbonate. These certified reference values (0.073 to 43.97 wt% CO_2) span the expected range of our unknows. Mean calibration inaccuracies (defined as certified reference value - measured value, reported in wt%) were less than ±0.01%. Prior to sample analysis, multiple blanks were analyzed with the accelerants added to crucibles. All blanks yield CO_2 concentrations <0.01 wt%.

Samples were run as unknowns and bracketed by internal standards to monitor accuracy and precision. Typically, six internal standards were bracketed at the start and end of 15 unknown analyses. Precision and accuracy of this method were assessed using internal standards composed of matrix-matched University of Southampton internal basalt standard BRR-1[2] and KEO-1, a sheeted dyke (diabase) from the Troodos ophiolite, Cyprus. Both internal standards yield low CO_2 contents (<0.1 wt%). Internal standards BRR-1 and KEO-1 were spiked with ultra-pure LECO calcium carbonate to produce additional internal standards BRR-1Y and KEO-1Y with 1.83 wt% CO_2 and BRR-1X and KEO1-X with 9.90 wt% CO_2, respectively, spanning the CO_2 concentration range of the rock samples. The mean internal precision (defined as 2 standard errors; 2SE) of BRR-1, BRR1-Y, and BRR-1X were 0.05 (n=7), 0.2 (n=8), and 0.42 (n=8) wt% CO_2, respectively, and KEO-1, KEO1-Y, and KEO-1X were 0.05 (n=8), 0.16 (n=8), and 0.38 (n=8) wt% CO_2, respectively. The long-term external precision (2SE) of BRR-1,

BRR1-Y, and BRR-1X were 0.09 (n=34), 0.18 (n=36), and 0.20 (n=36) wt% CO_2, respectively and KEO-1, KEO1-Y, and KEO-1X were 0.11 (n=34), 0.15 (n=34), and 0.37 (n=34) wt% CO_2, respectively. The mean CO_2 contents of BRR-1, BRR1-Y, and BRR-1X were 0.10 wt% (n=7), 1.85 wt% (n=8), and 10.07 wt% (n=8). This results in mean inaccuracies (defined as expected value - measured value, reported in wt%) for spiked standards BRR-1Y and BRR-1X of 0.02 and 0.17 wt% CO_2, respectively. The mean CO_2 contents of KEO-1, KEO1-Y, and KEO-1X were 0.06 wt% (n=8), 1.78 wt% (n=8), and 9.89 wt% (n=8), respectively. These yielded mean inaccuracies for spiked standards KEO-1Y and KEO-1X of 0.05 and 0.01 wt% $CO_{2,}$ respectively. Drift was monitored by analyzing drift standards (LECO 502-894 and 502-909) at the end of each session and were corrected using the LECO Cornerstone software. Data were reduced using the LECO Cornerstone software, with blank subtraction performed using full procedural blank crucibles. Carbon concentrations were converted to wt% CO_2 offline and are reported in Supplementary Table 2.

Carbonate samples representative of the different types of carbonate mineral occurrences in the Site U1557 breccias were selected for isotopic analysis. Carbonate material was extracted from each sample using a scalpel and handpicked to be optically clear, using a binocular microscope. Two samples were too hard for this method, and carbonate was extracted using a Dremel tool, instead. The clean carbonate fractions were powdered in an agate pestle and mortar. 20 mg of powdered sample was dissolved in 1 ml of 10% acetic acid and centrifuged before the supernatant was siphoned, removing any remaining non-carbonate solid material.

All samples were prepared for Sr isotope analysis at NOCS, where Sr was isolated using Strontium SpecTM columns and loaded onto Ta filaments (see[3] for details). $^{87}Sr/^{86}Sr$ ratios were

measured at the Bristol Isotope Group Facilities (University of Bristol) on a Thermo-Finnigan Triton thermal ionization mass spectrometer using a multi-dynamic 'triple jump' method.

Oxygen and carbon isotopic compositions were determined using a Thermo Scientific Kiel IV carbonate device coupled to a MAT253 isotope ratio mass spectrometer at NOCS. 20-40 µg of sample powder was weighed into borosilicate glass vials and reacted with 106% phosphoric acid at 70 °C for 600 seconds in the Keil IV. After cryogenic removal of water vapor and other gases, the resulting CO_2 was analyzed multiple times against a reference gas. Following data reduction and corrections, data were normalized using a two-point calibration with NBS 18 and NBS 19 (International Atomic Energy Agency, Vienna, Austria). A suitable in-house reference material (GS1, Carrara marble) was used for quality assurance purposes and to report instrument precision. Long-term instrument precision is 0.02‰ for $\delta^{13}C$ and 0.03‰ for $\delta^{18}O$. C and O isotopic compositions are reported using delta notation (as ‰ VPDB) in Supplementary Table 3.

Carbonate Precipitation Temperature Calculations

Carbonate precipitation temperatures (Supplementary Table 3) were calculated from the measured $\delta^{18}O$ of each carbonate sample using an experimentally determined oxygen isotope fractionation equation[4]:

$$\delta^{18}O_{CO3} - \delta^{18}O_{fluid} = 2.78 \times (10^6/T)^2 - 2.89 \qquad \text{(Eq. S1)}$$

where $\delta^{18}O_{CO3}$ and $\delta^{18}O_{fluid}$ are the oxygen isotopic compositions (expressed as $‰_{VSMOW}$) of the carbonate and fluid, respectively, and T is temperature in Kelvin. Following [5,6], we assume basement fluids have the oxygen isotopic composition of contemporaneous seawater as fluid-rock reaction on the young Juan de Fuca Ridge flank results in only a slight deviation in fluid

$\delta^{18}O$ from modern seawater values[7]. However, the oxygen isotopic composition of seawater has varied throughout Earth history and the exact timing of carbonate formation at Site U1557 is not known. To account for this, two endmember temperatures were calculated for each sample, one assuming an ice-free ocean with $\delta^{18}O_{VSMOW}$ of -1‰ and the other for an icehouse ocean with $\delta^{18}O_{VSMOW}$ of 0‰(8,9). To assess the potential ages of carbonate samples (i.e., ages at which each sample's precipitation temperature was consistent with formation from seawater or warmer basement fluids; Fig. 5) we use the "ice-free" endmember temperatures from 61 to 35 Ma when there were no polar ice sheets[8,9], and the "icehouse" end member temperatures since 15 Ma, when permanent polar ice sheets were established[10]. Between 35 and 15 Ma, when ice sheets were ephemeral[10], a given sample's precipitation temperature would fall somewhere between its "ice-house" and "ice-free" endmember temperatures (e.g., purple dashed lines in Fig. 5d).

To assess the uncertainty introduced by the choice of thermometer, four temperatures were calculated for each sample using a range of published inorganic carbonate thermometers (Supplementary Table 6; [4,11-13]). The online tool of reference [14] was used to calculate all but those based on reference [4]. For the purposes of this assessment fluid $\delta^{18}O$ was assumed to be -1‰$_{VSMOW}$. The standard deviation of the temperatures calculated with the four thermometers ranges from 0.82 to 1.55 °C, with the temperature calculated via reference [12] always appearing as an outlier. The thermometer of [11] is in good agreement with the others at warm (>6 °C) temperatures but at low temperatures it is far from its calibration range (14°C to 57 °C) resulting in divergence from the others and calculated water temperatures <0 °C. Very similar results are obtained assuming an icehouse ocean $\delta^{18}O$ of 0 ‰$_{VSMOW}$. Taking the mean standard deviation (0.57 °C) of the thermometers of [4,12,13], a conservative 2-sigma uncertainty of 1°C was applied to all calculated fluid temperatures (calculated using Equation S1(4)).

Deep Ocean Temperature Record

In Fig. 5b, we compare the calculated carbonate precipitation temperatures to seawater temperatures since 61 Ma to assess when the precipitation temperatures were consistent with formation from seawater or warmer ridge flank fluids. Benthic oxygen isotope records ($\delta^{18}O_{benthic}$) can be used to reconstruct deep sea temperatures, but only if the oxygen isotopic value of the original seawater is known. Here, we calculate deep ocean temperatures (T_{do}) using the most recent version of the deep sea benthic record[10] and the seawater $\delta^{18}O$ estimates of [15]. For the ice-free conditions between 66.0 and 34.2 Ma ($\delta^{18}O_{benthic}$ <1.75) we used the following equation from [15]:

$$T_{do}(°C) = 4\delta^{18}O_{benthic} + 12 \quad \text{(Eq. S2)}$$

For the coolhouse conditions prevailing between 34.2 and 3.64 Ma ($\delta^{18}O_{benthic}$ between 1.75 and 3.25), we used the following equation from [15]:

$$T_{do}(°C) = 5 - 8\frac{(\delta^{18}O_{benthic} - 1.75)}{3} \quad \text{(Eq. S3)}$$

For icehouse conditions prevailing after 3.65 Ma ($\delta^{18}O_{benthic}$ >3.25), we used the following equation from [15]:

$$T_{do}(°C) = 5 - 4.4\frac{(\delta^{18}O_{benthic} - 3.25)}{3} \quad \text{(Eq. S4)}$$

Supplementary Figures:

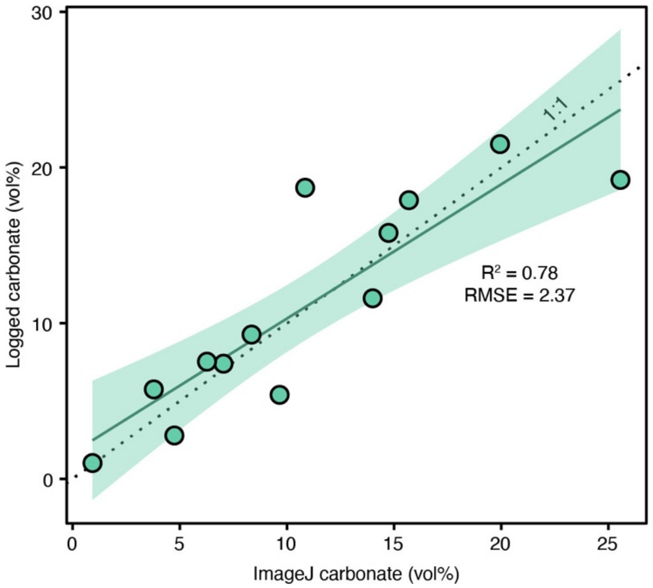

Supplementary Figure 1. Cross plot of the proportions of carbonate determined by visual core logging and image analysis in ImageJ for 13 selected core sections from Hole U1557D. Also shown is a dotted 1:1 line for comparison and a linear regression of the data with its associated 1σ confidence envelope. The R^2-value and root mean square error (RMSE) of this correlation are 0.78 and 2.37, respectively.

Supplementary Tables provided in additional files:

Supplementary Table 1 - ***see Excel workbook.***

Site U1557 core-logging results and CO_2 contents.

Volume abundance of each component (carbonate cement, altered basalt clasts, recrystallized pelagic sediment etc.) are given for each core. Their abundance in each core was calculated from shipboard logs made at the section or piece level, normalized to recovered rock using a piece log[1]. Original porosity is given as the sum of open porosity, cement (carbonate and authigenic),

recrystallized sediment, and non-carbonate secondary minerals. Core bulk densities and CO_2 contents are calculated following Equations 3 and 2, respectively. Carbonate cement is assumed to have a density of 2710 kg/m^3; *the densities of the other breccia components were determined from shipboard physical properties measurements of discrete samples[1]. The average abundance of each component at Site U1557 has been calculated as the mean weighted by the drilled interval of each core, to normalize for variable recovery. The average CO_2 content of the breccia deposit was calculated from core CO_2 contents, weighted by core interval lengths, to normalize for variable core recovery, following Equation 4.

Supplementary Table 2 - ***see Excel workbook.***

CO_2 concentrations of U1557 whole rock samples.

Supplementary Table 3 - ***see Excel workbook.***

Stable (C, O) and radiogenic (Sr) isotope compositions of carbonate samples.

b.d.l. = below detection limit. *For each sample, two precipitation temperatures have been calculated for end-member fluid δ^{18}O of -1‰ and 0‰ (reflecting the composition of seawater in ice-free and icehouse oceans, respectively) using the thermometer of [4].

Supplementary Table 4 - ***see Excel workbook.***

Breccia thickness model parameters and results.

Average breccia thickness (T_B) calculated using parametric bootstrapping with one million iterations over Equation 14, for sections of the Mid-Atlantic Ridge (MAR), Southern Explorer Ridge (SoExR), Cocos-Nazca Ridge (CoNz), and East Pacific Rise (EPR). The prior probability

distributions used are based on published observations of mean throw (t), total strain (ε), fault dip (F), and angle of repose (R) from each location, unless otherwise specified, using the following sources: MAR13°N [(16)]; MAR29°N [(17)]; SoExR [(18)]; CoNz85°W, EPR8.5-10°N, and RPR18-19°S [(19)]; EPR9.4-10°N [(20)]; EPR3-4°S [(21,22)]; EPR19°30'S [(23)]. We designate t, ε and F as exponential, uniform, and Gaussian distributions, respectively. [a] Where local observations of F are not reported, F is designated as a Gaussian distribution with a median value of 60°, a maximum dip angle of 75°, and minimum dip angle of 46° based on observed fault scarp geometries, and theoretical fault dips[24-27]. [b] Where local observations of the angle of repose are not reported, we use estimates based on observations of talus deposit geometries along the MAR north of the Kane Fracture Zone[28]. The proportion of the fault scarp covered by talus (x) is designated as 0.6 ± 0.1, based on observations of talus deposits along the MAR[29].

Supplementary Table 5 - *see Excel workbook.*

Validation of logged carbonate abundances.

Compared are abundances of open-space filling carbonate cement determined by core logging and by manual thresholding of grayscale images in ImageJ for 13 representative core sections covering the range of carbonate abundances.

Supplementary Table 6 - *see Excel workbook.*

Comparison of inorganic carbonate oxygen isotope thermometers.

Four published carbonate thermometers are compared for each of the carbonate samples in Supplementary Table 3. Carbonate oxygen isotopic compositions ($\delta^{18}O_{CO3}$; ‰ VPBD unless

specified) were converted to temperature (T; °C) as follows, assuming precipitation from fluid with the composition of seawater in an ice-free world ($\delta^{18}O_{fluid}$ = -1‰$_{VSMOW}$):

a. using the inorganic calibration of [13] with equation:

$$T = 16.1 - 4.64(\delta^{18}O_{CO3} - \delta^{18}O_{fluid} - 0.27) + 0.09(\delta^{18}O_{CO3} - \delta^{18}O_{fluid} - 0.27)$$

Temperatures were calculated via the online tool of [14], with an offset of -0.27‰ applied to convert VSMOW to VPBD following the method used to construct the original calibration [30].

b. using the inorganic calibration of [12] with equation:

$$T = 16.9 - 4.38(\delta^{18}O_{CO3} - \delta^{18}O_{fluid} - 0.20) + 0.10(\delta^{18}O_{CO3} - \delta^{18}O_{fluid} - 0.20)$$

Temperatures were calculated via the online tool of [14], with an offset of -0.20‰ applied to convert VSMOW to VPBD, following the method used to construct the original calibration [31].

c. using the inorganic calibration of [11] with equation:

$$T = 16.0 - 5.17(\delta^{18}O_{CO3} - \delta^{18}O_{fluid} - 0.20) + 0.09(\delta^{18}O_{CO3} - \delta^{18}O_{fluid} - 0.20)$$

Temperatures were calculated via the online tool of [14], with an offset of -0.20‰ applied to convert VSMOW to VPBD, following the method used to construct the original calibration [31].

d. using the inorganic calibration of [4] with equation:

$$T = ((2.78\times10^{6}) / (\delta^{18}O_{CO3}[VSMOW] - \delta^{18}O_{fluid} + 2.89))^{0.5} - 273.15$$

Supplementary References:

1 Coggon, R. M. *et al. Proceedings of the International Ocean Discovery Program*. Vol. 390/393 (International Ocean Discovery Program, 2024).

2 Taylor, R. N., Murton, B. J. & Thirwall, M. F. Petrographic and geochemical variation along the Reykjanes Ridge, 57°N–59°N. *Journal of the Geological Society* **152**, 1031-1037, doi:10.1144/GSL.JGS.1995.152.01.25 (1995).

3 Harris, M. *et al.* Channelling of hydrothermal fluids during the accretion and evolution of the upper oceanic crust: Sr isotope evidence from ODP Hole 1256D. *Earth Planet. Sci. Lett.* **416**, 56-66, doi:http://dx.doi.org/10.1016/j.epsl.2015.01.042 (2015).

4 Friedman, I. & O'Neil, J. R. Compilation of stable isotope fractionation factors of geochemical interest in *Data of Geochemistry, United States Geological Survey Professional Paper 440-KK* (ed M. Fleischer) 1-12 (USGS, 1977).

5 Coggon, R. M., Teagle, D. A. H., Cooper, M. J. & Vanko, D. A. Linking basement carbonate vein compositions to porewater geochemistry across the eastern flank of the Juan de Fuca Ridge, ODP Leg 168. *Earth Planet. Sci. Lett.* **219**, 111-128, doi:https://doi.org/10.1016/S0012-821X(03)00697-6 (2004).

6 Coggon, R. M., Teagle, D. A. H., Smith-Duque, C. E., Alt, J. C. & Cooper, M. J. Reconstructing past seawater Mg/Ca and Sr/Ca from mid-ocean ridge flank calcium carbonate veins. *Science* **327**, 1114-1117, doi:https://doi.org/10.1126/science.1182252 (2010).

7 Elderfield, H., Wheat, C. G., Mottl, M. J., Monnin, C. & Spiro, B. Fluid and geochemical transport through oceanic crust: a transect across the eastern flank of the Juan de Fuca

Ridge. *Earth Planet. Sci. Lett.* **172**, 151-165, doi:https://doi.org/10.1016/S0012-821X(99)00191-0 (1999).

8 Meckler, A. N. *et al.* Cenozoic evolution of deep ocean temperature from clumped isotope thermometry. *Science* **377**, 86-90, doi:https://doi.org/10.1126/science.abk0604 (2022).

9 Cramer, B. S., Miller, K. G., Barrett, P. J. & Wright, J. D. Late Cretaceous-Neogene trends in deep ocean temperature and continental ice volume: Reconciling records of benthic foraminiferal geochemistry (delta^{18}O and Mg/Ca) with sea level history. *Journal of Geophysical Research (Oceans)* **116**, C12023, doi:https://doi.org/10.1029/2011jc007255 (2011).

10 Westerhold, T. *et al.* An astronomically dated record of Earth's climate and its predictability over the last 66 million years. *Science* **369**, 1383-1387, doi:https://doi.org/10.1126/science.aba6853 (2020).

11 McCrea, J. M. On the isotopic chemistry of carbonates and a paleotemperature scale. *J. Chem. Phys.* **18**, 849-857, doi:https://doi.org/10.1063/1.1747785 (1950).

12 O'Neil, J. R., Clayton, R. N. & Mayeda, T. K. Oxygen isotope fractionation in divalent metal carbonates. *J. Chem. Phys.* **51**, 5547-5558, doi:https://doi.org/10.1063/1.1671982 (1969).

13 Kim, S.-T. & O'Neil, J. R. Equilibrium and nonequilibrium oxygen isotope effects in synthetic carbonates. *Geochim. Cosmochim. Acta* **61**, 3461-3475, doi:https://doi.org/10.1016/S0016-7037(97)00169-5 (1997).

14 Gaskell, D. E. & Hull, P. M. delta^{18}O to temperature converter https://research.peabody.yale.edu/d180/index.html (2023).

15 Hansen, J., Sato, M., Russell, G. & Kharecha, P. Climate sensitivity, sea level and atmospheric carbon dioxide. *Philos. Trans. R. Soc. London, A* **371**, 20120294, doi:https://doi.org/10.1098/rsta.2012.0294 (2013).

16 MacLeod, C. J. *et al.* Life cycle of oceanic core complexes. *Earth Planet. Sci. Lett.* **287**, 333-344 (2009).

17 Escartin, J. *et al.* Quantifying tectonic strain and magmatic accretion at slow spreading ridge segment, Mid-Atlantic Ridge, 29°N. *J. Geophys. Res.* **104**, 10421-10437, doi:https://doi.org/10.1029/1998JB900097 (1999).

18 Deschamps, A., Tivey, M., Embley, R. W. & Chadwick, W. W. Quantitative study of the deformation at Southern Explorer Ridge using high-resolution bathymetric data. *Earth Planet. Sci. Lett.* **259**, 1-17, doi:https://doi.org/10.1016/j.epsl.2007.04.007 (2007).

19 Carbotte, S. & MacDonald, K. C. Comparison of seafloor tectonic fabric at intermediate, fast, and super fast spreading ridges: Influence of spreading rate, plate motions, and ridge segmentation on fault patterns. *Journal of Geophysical Research: Solid Earth* **99**, 13609-13631, doi: https://doi.org/10.1029/93JB02971 (1994).

20 Escartín, J. *et al.* Interplay between faults and lava flows in construction of the upper oceanic crust: The East Pacific Rise crest 9°25′–9°58′N. *Geochemistry, Geophysics, Geosystems* **8**, doi:https://doi.org/10.1029/2006GC001399 (2007).

21 Searle, R. Gloria survey of the east pacific rise near 3.5°S: Tectonic and volcanic characteristics of a fast spreading mid-ocean rise. *Tectonophysics* **101**, 319-344, doi:https://doi.org/10.1016/0040-1951(84)90119-7 (1984).

22 Cowie, P. A., Scholz, C. H., Edwards, M. & Malinverno, A. Fault strain and seismic coupling on mid-ocean ridges. *Journal of Geophysical Research: Solid Earth* **98**, 17911-17920, doi:https://doi.org/10.1029/93JB01567 (1993).

23 Bohnenstiehl, D. R. & Carbotte, S. M. Faulting patterns near 19°30′S on the East Pacific Rise: Fault formation and growth at a superfast spreading center. *Geochemistry, Geophysics, Geosystems* **2**, doi:https://doi.org/10.1029/2001GC000156 (2001).

24 Anderson, E. M. The dynamics of faulting. *Transactions of the Edinburgh Geological Society* **8**, 387-402, doi:https://doi.org/10.1144/transed.8.3.387 (1905).

25 Sibson, R. H. Frictional constraints on thrust, wrench and normal faults. *Nature* **249**, 542-544, doi:10.1038/249542a0 (1974).

26 Collettini, C. & Sibson, R. H. Normal faults, normal friction? *Geology* **29**, 927-930, doi:10.1130/0091-7613(2001)029<0927:Nfnf>2.0.Co;2 (2001).

27 Chen, J., Escartin, J. & Cannat, M. Fault scarps and tectonic strain in young volcanic seafloor. *Earth Planet. Sci. Lett.* **651**, 119174, doi:https://doi.org/10.1016/j.epsl.2024.119174 (2025).

28 Allerton, S., Murton, B. J., Searle, R. C. & Jones, M. Extensional faulting and segmentation of the Mid-Atlantic Ridge north of the Kane Fracture Zone (24°00' N to 24°40' N). *Marine Geophysical Researches* **17**, 37-61, doi:https://doi.org/10.1007/BF01268050 (1995).

29 Cannat, M., Mangeney, A., Ondréas, H., Fouquet, Y. & Normand, A. High-resolution bathymetry reveals contrasting landslide activity shaping the walls of the Mid-Atlantic Ridge axial valley. *Geochem. Geophys. Geosys.* **14**, 996-1011, doi:https://doi.org/10.1002/ggge.20056 (2013).

30 Hut, G. Consultants' group meeting on stable isotope reference samples for geochemical and hydrological investigations. Report No. INIS-MF--10954, (International Atomic Energy Agency, 1987).

31 Bemis, B. E., Spero, H. J., Bijma, J. & Lea, D. W. Reevaluation of the oxygen isotopic composition of planktonic foraminifera: Experimental results and revised paleotemperature equations. *Paleoceanography* **13**, 150-160, doi:https://doi.org/10.1029/98PA00070 (1998).

List of the South Atlantic Transect IODP Expedition 390 & 393 Scientists:

Rosalind M. Coggon[1], Damon A. H. Teagle[1], Jason B. Sylvan[2], Julia S. Reece[3], Emily R. Estes[4,5], Trevor Williams[4], Gail L. Christeson[5], Masataka Aizawa[6], Elmar J. Albers[7], Chiara Amadori[8], Thomas M. Belgrano[9], Chiara Borrelli[10], Joshua D. Bridges[10], Elliot J. Carter[11], Timothy D'Angelo[12], Jaume Dinarès-Turell[13], Nobuhiro Doi[14], Justin Estep[15], Aled D. Evans[1], Marlo Garnsworthy[16], William P. Gilhooly[17], Lewis J. C. Grant[1], Gilles Guérin[18], Laura Guertin[19], Michelle Harris[8], Victoria Hojnacki[20], Gilbert Hong[21], Xiaobo Jin[22], Mallika Jonnalagadda[23], Michael R. Kaplan[18], Pamela D. Kempton[24], Walter Kurz[25], Daisuke Kuwano[26], Jessica M. Labonté[27], Adriane R. Lam[28], Marcin Latas[29], Christopher M. Lowery[30], Wanyi Lu[31], Andrew McIntyre[32], Paul Moal-Darrigade[33], Tessa Lund Peixoto[34], Stephen F. Pekar[35], Muthusamy Prakasam[36], Claudio Robustelli Test[37], Claire M. Routledge[38], Jeffrey G. Ryan[39], Danielle P. Santiago Ramos[40], Alina Shchepetkina[41], Angela L. Slagle[18], Mako Takada[42], Leonardo Tamborrino[43], Liyan Tian[44], Alexandra Villa[43], Yi Wang[45], ShuYing Wee[2], Sarah Widlansky[46], Kiho Yang[47], Tiantian Yu[48], Guoliang Zhang[49].

Affiliations:

[1] School of Ocean and Earth Science, National Oceanography Centre Southampton, University of Southampton; Southampton, SO14 3ZH, UK.

[2] Department of Oceanography, Texas A&M University; College Station TX 77843, USA.

[3] Department of Geology & Geophysics, Texas A&M University; College Station TX 77843, USA.

[4] International Ocean Discovery Program, Texas A&M University; College Station TX 77845, USA.

[5] Marine Geology and Geophysics, National Science Foundation; Alexandria VA 22314, USA.

[6] Graduate School of Engineering Science, Akita University; Akita 010-8502, Japan

[7] Section of Geophysics, Alfred Wegener Institute, Helmholtz Centre for Polar and Marine Research; 27570 Bremerhaven, Germany.

[8] School of Geography, Earth and Environmental Sciences, University of Plymouth; Plymouth, PL4 8AA, UK.

[9] UCD School of Earth Sciences, University College Dublin, Dublin, Ireland

[10] Department of Earth and Environmental Sciences, University of Rochester; Rochester NY 14627, USA.

[11] School of Life Sciences, Keele University, Newcastle-under-Lyme, ST5 5BG, UK

[12] Bigelow Laboratory for Ocean Sciences; East Boothbay ME 04544, USA.

[13] Istituto Nazionale di Geofisica e Vulcanologia; Rome, 00143 Rome, Italy.

[14] Division of Earth and Environmental Sciences, Chiba University; Chiba 263-8522, Japan.

[15] School of Earth and Sustainability, Northern Arizona University; Flagstaff, AZ 86011, USA.

[16] Icebird Studio; USA.

[17] Department of Earth and Environmental Sciences, Indiana University Indianapolis; Indianapolis, IN 46202, USA.

[18] Lamont-Doherty Earth Observatory, Columbia University; Palisades, NY 10964, USA.

[19] Earth Science, Penn State Brandywine; Media PA 19063, USA.

[20] School of Natural Sciences, Mercyhurst University, Erie, PA 16546, USA

[21] School of Earth and Environmental Sciences, Seoul National University; Seoul, Republic of Korea.

[22] School of Ocean and Earth Science, Tongji University; Shanghai, China.

[23] Interdisciplinary School of Science Savitribai Phule, Pune University; Pune, Maharashtra 411007, India.

[24] Department of Geology, Kansas State University; Manhattan, KS 66506, USA.

[25] Institute of Earth Sciences, University of Graz; Graz, 8010 Graz, Austria.

[26] Graduate School of Human and Environmental Studies, Kyoto University; Kyoto, 606-8501, Japan.

[27] Department of Marine Biology, Texas A&M University at Galveston; Galveston, TX 77554, USA.

[28] Earth Sciences Department, Binghamton University; Binghamton, NY 13902, USA.

[29] School of Geography, Earth and Environmental Sciences, University of Birmingham, Birmingham, B15 2TT, UK

[30] Institute for Geophysics, University of Texas at Austin; Austin, TX 78758, USA.

[31] Department of Geology & Geophysics, Woods Hole Oceanographic Institution; Woods Hole (MA), USA.

[32] School of Geography, Geology and the Environment, University of Leicester; Leicester, LE1 7RH, UK.

[33] UMR EPOC Oceanic and Continental Environments and Paleoenvironments, University of Bordeaux; 33000 Bordeaux, France.

[34] JVS Boston, Boston; MA 02110, USA.

[35] School of Earth and Environmental Sciences, Queens College (CUNY); Queens, NY 11367, USA.

[36] Wadia Institute of Himalayan Geology; Uttarakhand, 248001, India.

[37] Department of Earth Sciences, University of Turin; 10124 Turin, Italy.

[38]Institute of Geosciences, Christian-Albrechts-University of Kiel; 24118 Kiel, Germany.

[39] School of Geosciences, University of South Florida; Tampa, FL 33620, USA.

[40] Department of Marine and Coastal Sciences Rutgers University; New Brunswick, NJ 08901, USA.

[41] Department of Earth and Atmospheric Sciences, University of Western Ontario; London, ON N6A 5B7, Canada.

[42] Graduate School of Frontier Sciences, University of Tokyo; Chiba, 277-0882, Japan.

[43] MARUM—Center for Marine Environmental Sciences, University of Bremen; 28359 Bremen, Germany.

[44] Institute of Deep-sea Science and Engineering, Chinese Academy of Sciences; Sanya, 572000, China.

[45] Department of Earth and Environmental Sciences, Tulane University; New Orleans, LA, USA, 70118.

[46] Woods Hole Coastal and Marine Science Center, U.S. Geological Survey; Woods Hole, MA 02543.

[47] Department of Oceanography and Marine Research Institute, Pusan National University; Busan, 46241, Republic of Korea.

[48] School of Oceanography, Shanghai Jiao Tong University; Xuhui District, Shanghai, China.

[49] Institute of Oceanology, Chinese Academy of Sciences; Guangzhou Yuexiu District 510070, China.
